# ADISSP Drives Metabolic Reprogramming and Immune Suppression to Regulate Hepatocellular Carcinoma Progression and Immunotherapy Sensitivity

**DOI:** 10.1002/advs.78582

**Published:** 2026-11-10

**Authors:** Tuan‐Song Zhang, Chen Xiong, Chen Yang, Yu‐Chuan Yan, Jie Li, Rui‐Zhe Li, Guo‐Qiang Pan, Jie Zou, Xiao‐Lu Zhang, Tao Li, Zhao‐Ru Dong

**Affiliations:** ^1^ Shandong Key Laboratory of Malignant Tumor Precision Treatment, Department of General Surgery Qilu Hospital, Shandong University Jinan China; ^2^ Research Center for Basic Medical Sciences Qilu Hospital, Shandong University Jinan China; ^3^ Department of Geriatrics Qilu Hospital, Shandong University Jinan China; ^4^ Department of Physiology and Pathophysiology School of Basic Medical Sciences Cheeloo College of Medicine Shandong University Jinan Shandong China

**Keywords:** ADISSP, ferroptosis, HCC, macrophage polarization, NF‐κB pathway, PPP1CA

## Abstract

Metabolic reprogramming and immune evasion are critical drivers of hepatocellular carcinoma (HCC) progression and therapeutic resistance, yet the upstream regulators linking these processes remain poorly understood. Here, we identify adipogenesis‐associated secreted signaling protein (ADISSP) as a previously unrecognized oncogenic regulator in HBV‐related HCC. ADISSP was markedly upregulated in HCC tissues and associated with poor prognosis, while its inhibition suppressed tumor progression in vitro and in vivo. Mechanistically, ADISSP interacted with PPP1CA to sustain NF‐κB activation, resulting in transcriptional induction of SLC16A3 and CCL20. The ADISSP/SLC16A3 axis promoted glycolytic adaptation and lactate‐mediated STAT3 activation, thereby driving macrophage M2 polarization, whereas CCL20 facilitated macrophage recruitment. Together, these pathways established an immunosuppressive tumor microenvironment characterized by enhanced M2 macrophage infiltration and impaired antitumor immunity. More important, targeting ADISSP markedly enhanced the therapeutic efficacy of anti‐PD‐1 plus lenvatinib in HCC. Collectively, our findings identify ADISSP as a central regulator integrating metabolic reprogramming and immune suppression through the PPP1CA/NF‐κB axis and highlight ADISSP as a potential therapeutic target for increasing immunotherapy sensitivity in HCC.

## Graphical abstract

In HCC, ADISSP sustains IKKβ/NF‐κB signaling by inhibiting PPP1CA‐mediated IKKβ dephosphorylation, thereby inducing the expressions of SLC16A3 and CCL20. SLC16A3‐driven lactate remodeling promotes glycolysis, ferroptosis resistance, and M2 macrophage polarization, whereas CCL20 facilitates macrophage recruitment. More important, targeting ADISSP markedly enhanced the therapeutic efficacy of anti‐PD‐1 plus lenvatinib for HCC.

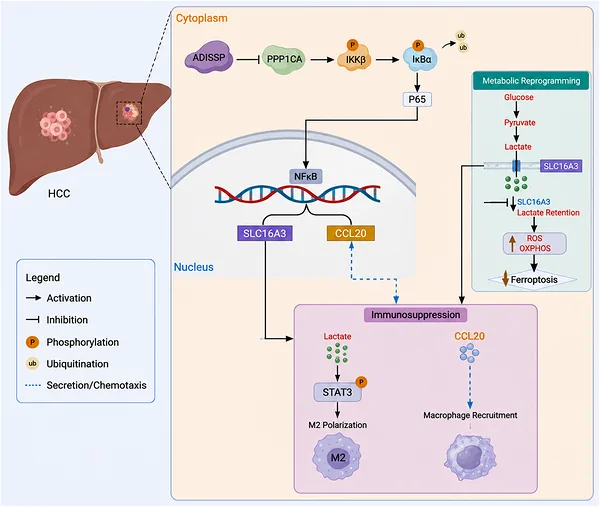

**Abbreviations**
ADISSP: adipose secreted signaling protein
CCL20: C‐C motif chemokine ligand 20
ChIP: chromatin immunoprecipitation
DEGs: differentially expressed genes
DFS: disease‐free survival
ECAR: extracellular acidification rate
GO: Gene Ontology
HBV: hepatitis B virus
HCC: hepatocellular carcinoma
ICB: immune checkpoint blockade
KEGG: Kyoto Encyclopaedia of Genes and Genomes
MCT: monocarboxylate transporters
mRNA: messenger RNA
NASH: non‐alcoholic steatohepatitis
NF‐κB: nuclear factor‐κB
OCR: oxygen consumption rate
OS: overall survival
PD1: programmed cell death 1
PMA: phorbol‐12‐myristate‐13‐acetate
PP1: protein phosphatase 1
PPP1CA: protein phosphatase 1 catalytic subunit alpha
SLC16A3: solute carrier family 16 member 3
TAMs: tumor‐associated macrophages
TCGA: The Cancer Genome Atlas
TIME: tumor immune microenvironment

## 1 Introduction

Hepatocellular carcinoma (HCC) remains one of the leading causes of cancer‐related mortality worldwide and represents a major global health burden [1]. Despite substantial advances in early diagnosis and therapeutic strategies, the prognosis of patients with advanced HCC remains poor [2]. In recent years, immune checkpoint blockade (ICB) combined with tyrosine kinase inhibitors (TKIs), such as anti‐PD‐1 therapy plus lenvatinib, has significantly improved the treatment landscape for advanced HCC [3, 4]; however, durable clinical responses are achieved only in a limited proportion of patients [5]. The emergence of primary and acquired therapeutic resistance remains a major obstacle to improving patient outcomes. Increasing evidence indicates that treatment resistance is driven by complex interactions between tumor‐intrinsic alterations and the tumor microenvironment (TME), among which metabolic adaptation and immune suppression represent two fundamental features of malignant progression [6, 7, 8]. Tumor cells undergo extensive metabolic rewiring to sustain proliferation, survive therapeutic stress, and reshape the surrounding immune landscape [9, 10]. However, the molecular mechanisms that coordinately regulate metabolic plasticity and immune escape in HCC remain incompletely understood.

Aerobic glycolysis is a well‐established hallmark of HCC and plays a critical role in supporting tumor growth and adaptation under metabolic stress [11]. Beyond serving as a metabolic substrate, glycolysis‐derived metabolites have emerged as important signaling molecules that actively regulate tumor–immune interactions [12]. Lactate, the major end product of aerobic glycolysis, accumulates within the tumor microenvironment and exerts profound immunomodulatory effects [13, 14]. Tumor‐derived lactate not only facilitates cancer cell survival but also promotes macrophage polarization toward an immunosuppressive M2‐like phenotype, impairs cytotoxic T‐cell function, and contributes to immune escape [15, 16]. In parallel, chemokine‐mediated recruitment of immunosuppressive myeloid populations further reinforces tumor immune tolerance and limits the efficacy of immunotherapy [17]. These findings highlight the close relationship between tumor metabolism and immune regulation. Nevertheless, metabolic remodeling and immune suppression have largely been investigated as independent processes, and the upstream molecular regulators that integrate these two biological programs in HCC remain poorly defined. Identifying such regulators may provide new insights into tumor progression and reveal therapeutic vulnerabilities for improving immunotherapy efficacy.

Nuclear factor‐κB (NF‐κB) signaling is a central transcriptional pathway involved in diverse biological processes, including inflammation, metabolism, cell survival, and immune regulation [18]. Aberrant activation of NF‐κB has been extensively implicated in HCC development by promoting malignant phenotypes, inflammatory responses, and immune evasion. In addition to regulating classical inflammatory mediators, NF‐κB directly controls the transcription of numerous genes involved in metabolic adaptation, cytokine secretion, and immune cell recruitment, suggesting that this pathway may serve as a molecular bridge connecting tumor metabolism with immune remodeling [19]. However, although NF‐κB activation is frequently observed in HCC, the upstream mechanisms responsible for its persistent activation remain incompletely understood. Moreover, the downstream transcriptional programs through which NF‐κB simultaneously regulates tumor metabolic adaptation and immune suppression require further investigation. Therefore, identifying novel regulators controlling NF‐κB activity may provide important insights into how malignant cells coordinate intrinsic metabolic changes with extrinsic immune modulation.

ADISSP (adipogenesis‐associated secreted signaling protein) is a recently identified protein initially characterized in adipogenic processes, while its biological function in cancer remains largely unexplored [20]. Emerging evidence suggests that ADISSP may participate in metabolic regulation [21]; however, whether ADISSP contributes to tumor progression, immune remodeling, or therapeutic resistance has not been investigated. In this study, we identify ADISSP as a previously unrecognized oncogenic regulator in HBV‐related HCC. We demonstrate that ADISSP directly interacts with PPP1CA and suppresses PPP1CA‐mediated IKKβ dephosphorylation, thereby sustaining NF‐κB activation. Activated NF‐κB transcriptionally induces the expressions of SLC16A3 and CCL20, linking tumor metabolic reprogramming with immune microenvironment remodeling. Through the SLC16A3‐lactate pathway, ADISSP promotes glycolytic adaptation and macrophage M2 polarization, whereas CCL20 facilitates macrophage recruitment into the tumor microenvironment. Furthermore, we find that targeting ADISSP enhances the therapeutic efficacy of anti‐PD‐1 plus lenvatinib treatment by restoring antitumor immunity. Collectively, our findings establish ADISSP as a central regulator integrating metabolic reprogramming and immune suppression and highlight ADISSP inhibition as a promising therapeutic strategy for improving immunotherapy responses in HBV‐related HCC.

## 2 Results

### 2.1 Targeting Abnormally Elevated ADISSP Inhibits HCC Progression In Vivo and In Vitro

We started our study to screen for potential targets that might be associated with HBV‐related HCC progression. Our study performed an integrated analysis of HBV‐related HCC GEO databases, including GSE105130, GSE122482, GSE65485, and the HBV‐related TCGA‐LIHC dataset [22, 23, 24]. Then, we analyzed the profile of sorafenib‐resistant HCC cells (GSE109211) using the similar approach [25]. Transcriptomic analysis showed that the expressions of 38 genes were commonly increased in human HBV‐related HCC samples (Figure 1A). Among these 38 candidates, the five genes with the most significant expression differences between tumor and normal tissues were selected for visualization in a heatmap (Figure 1B). Kaplan–Meier analysis of the TCGA database showed that elevated ADISSP expression was significantly associated with poor overall survival (OS) and disease‐free survival (DFS) in patients with early HBV‐related HCC (Figure 1C). Next, we verified the higher ADISSP mRNA and protein levels in HBV‐related HCC tissues than in adjacent liver tissues, and ADISSP expression in tumor tissues was correlated with a worse prognosis (Figure 1D–F). TMA clinical data revealed a statistically significant correlation between ADISSP levels and several clinicopathological parameters, including tumor diameter, tumor capsule status, tumor number, and serum AFP levels in HBV‐related HCC patients (Table S4). Univariate and multivariate analyses identified ADISSP expression as an independent prognostic factor for OS and RFS in HBV‐related HCC patients (Tables S5 and S6). Furthermore, we evaluated whether ADISSP expression pattern was correlated with oncogenic mutation profiles. No significant differences in ADISSP expression were detected between CTNNB1‐mutant and CTNNB1‐wild‐type, TP53‐mutant and TP53‐wild‐type, or ARID1A‐mutant and ARID1A‐wild‐type tumors (Figure S1A). Those findings showed that ADISSP might act as a potential oncogene in HBV‐related HCC independent of standard oncogenic mutation profiles.

**FIGURE 1:**
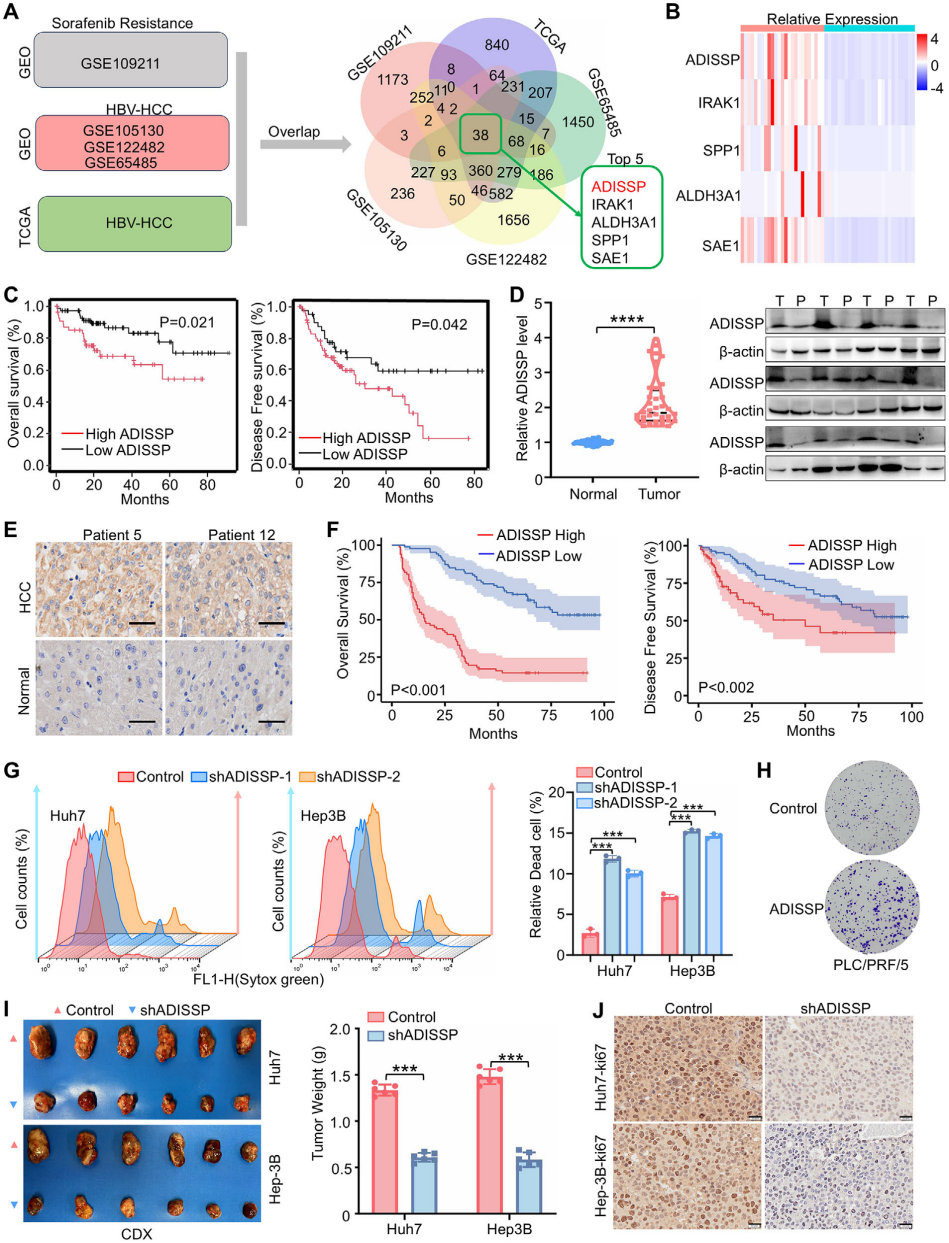
High ADISSP expression promotes HCC progression in vitro and in vivo. (A) Integrated screening strategy using several GEO datasets and the HBV‐stratified TCGA‐LIHC cohort, yielding 38 overlapping candidate genes. (B) Heatmap of five candidate genes. (C) Kaplan–Meier analysis demonstrated that ADISSP was predictive of OS and DFS in HCC patients. Patients were stratified into ADISSP‐high and ADISSP‐low groups using the optimal cutoff determined by Kaplan–Meier Plotter. (D) qRT–PCR and Western blotting (WB) revealed that ADISSP expression was significantly upregulated in paired HCC tissue samples. T, tumor; P, peritumoral. (E) IHC staining revealed that the protein expression of ADISSP in HBV‐related HCC tissue was significantly greater than that in normal liver tissue. Scale bars, 20 µm. (F) Kaplan–Meier analysis of OS and DFS in 167 HBV‐positive HCC patients stratified by median ADISSP expression (high, > median, *n* = 82; low, ≤ median, *n* = 85). High ADISSP expression was associated with poorer OS and DFS. (G) Flow cytometry analysis revealed that cell death significantly increased after the inhibition of ADISSP expression. Mean ± SD, *n* = 3, Student's *t*‐test, ^*^ *p* < 0.05, ^**^ *p* < 0.01, ^***^ *p* < 0.001. (H) Colony formation assay demonstrating the clonogenic ability of HCC cells. (I) Xenograft tumours derived from Huh7‐KD cells and Hep3B‐KD cells were significantly smaller than those derived from the corresponding control cells. Mean ± SD, *n* = 6, Student's *t*‐test, ^*^ *p* < 0.05, ^**^ *p* < 0.01, ^***^ *p* < 0.001. (J) An IHC assay of Ki67 revealed that inhibition of ADISSP inhibited tumour proliferation in vitro. Scale bars, 20 µm.

Then, we attempted to determine whether ADISSP dysregulation is broadly observed across other HCC main subtypes. To address this issue, we extended our analysis to independent GEO cohorts representing other major HCC etiologies. In the HCV‐associated HCC cohort (GSE17856), a statistically significant difference in ADISSP expression was detected, but the magnitude of change was minimal (log_2_FC = 0.079) (Figure S1B). In alcohol‐related HCC (GSE59259), ADISSP showed a modest increase (log_2_FC = 0.793) (Figure S1C). In MASH‐associated HCC cohorts (GSE99807, GSE164760 and GSE192959), no statistically significant alteration in ADISSP expression was observed (Figure S1D). Those results suggested that ADISSP may also has promoting effect on alcohol‐related HCC progression.

To determine the biological function of ADISSP, we first examined its endogenous expression in four HCC cell lines, including Huh7, Hep3B, PLC/PRF/5 and MHCC97H. Based on the relative expression levels, lentivirus‐mediated transduction was used to generated stable ADISSP‐knockdown and ADISSP‐overexpressing cell lines, designated Hep3B‐KD (ADISSP‐knockdown Hep3B cells), Huh7‐KD (ADISSP‐knockdown Huh7 cells) and PLC/PRF/5‐OE (ADISSP‐overexpressing PLC/PRF/5 cells) (Figure S2A–C). ADISSP knockdown significantly reduced the proliferation, invasion, and migratory capacities of Hep3B and Huh7 cells, whereas ADISSP over‐expression markedly enhanced these malignant phenotypes in PLC/PRF/5 cells. Furthermore, ADISSP inhibition significantly increased cell death (Figure 1G,H and Figure S2D–G). Xenograft models revealed a significant reduction in tumour size in both the Hep3B‐KD and Huh7‐KD groups compared with their respective controls (Figure 1I). Immunohistochemical (IHC) analysis revealed significantly lower expressions of Ki67 in the ADISSP‐KD groups than in the control groups (Figure 1J). Those findings demonstrate that ADISSP inhibition suppresses HCC progression in vitro and in vivo.

### 2.2 ADISSP Activates NF‐κB Signaling to Transcriptionally Upregulate SLC16A3 and CCL20 Expressions in HCC

To elucidate the molecular mechanisms underlying the oncogenic role of ADISSP in HCC, RNA sequencing was performed in stable ADISSP‐knockdown Hep3B and Huh7 cells, with their corresponding control cells (NC). Transcriptomic profiling identified 683 and 593 differentially expressed genes (DEGs) in Hep3B and Huh7 cells, respectively, among which 413 DEGs were commonly altered in both cell lines (Figure 2A and Figure S3A). Gene Ontology (GO) enrichment analysis revealed that these shared DEGs were predominantly enriched in glycolysis‐related metabolic processes and immune‐associated biological pathways. Consistently, Kyoto Encyclopedia of Genes and Genomes (KEGG) pathway analysis identified the NF‐κB signaling pathway as the most significantly enriched pathway (Figure 2B). We further confirmed the markedly reduced phosphorylation of p65 and IκBα in ADISSP‐KD HCC cells, whereas ADISSP overexpression produced the opposite effects in PLC/PRF/5 cells (Figure 2C). ADISSP depletion significantly impaired p65 nuclear translocation (Figure 2D,E and Figure S3B). To further determine whether NF‐κB activation is functionally required for ADISSP‐mediated oncogenic effects, ADISSP‐depleted cells were treated with TNF‐α, a canonical activator of the NF‐κB pathway. TNF‐α treatment restored the phosphorylation of p65 and IκBα (Figure 2F) and largely rescued the impaired proliferative, migratory, and invasive capacities induced by ADISSP depletion (Figure S3C,D). Conversely, pharmacological inhibition of p65 in PLC/PRF/5‐OE cells markedly reduced p65 phosphorylation and abolished the enhanced proliferation, migration, and invasion mediated by ADISSP overexpression (Figure 2G and Figure S3E,F). These findings establish NF‐κB signaling as a critical downstream effector of ADISSP in HCC cells.

**FIGURE 2:**
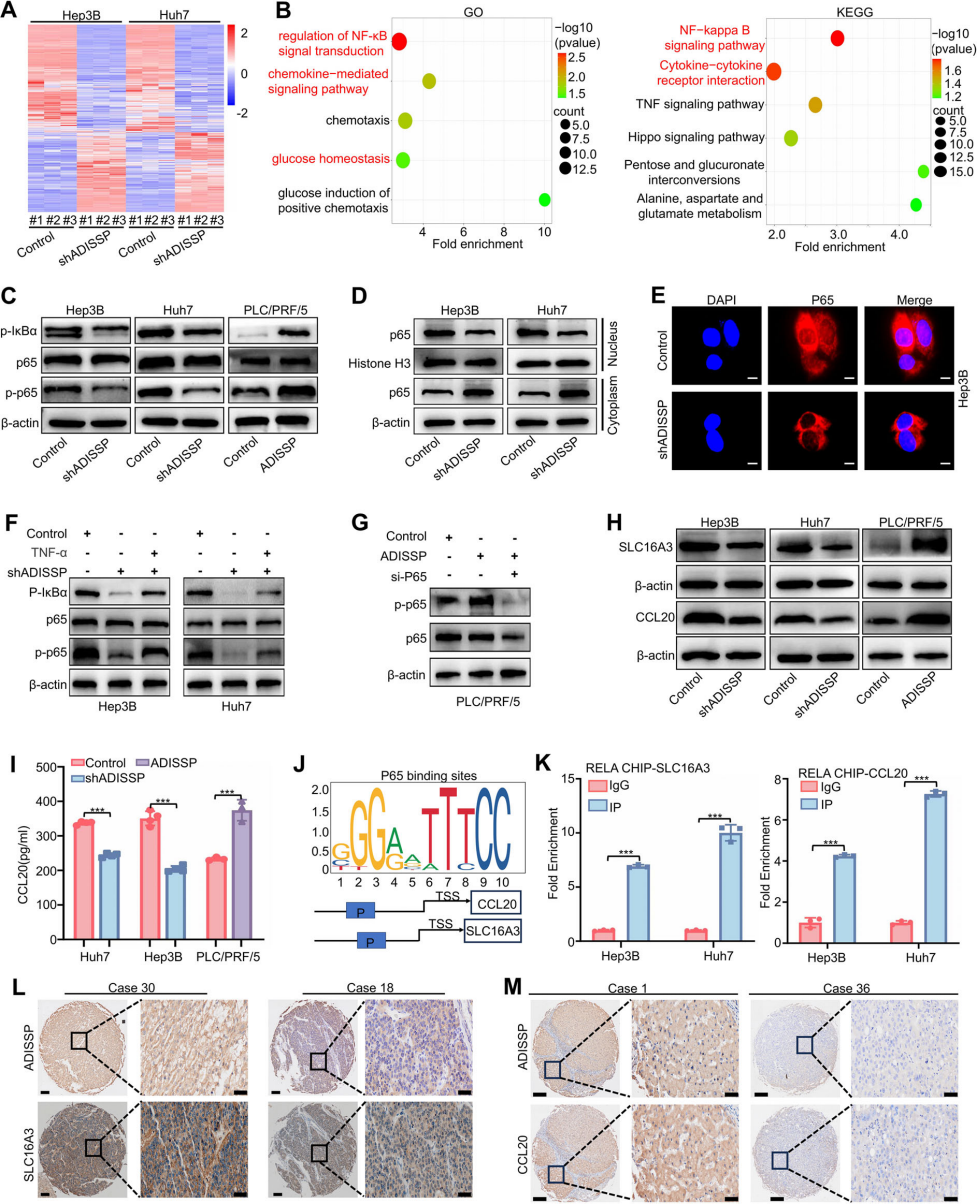
ADISSP activates NF‐κB signaling to transcriptionally upregulate SLC16A3 and CCL20 expressions in HCC. (A) Heatmap of the DEGs between Huh7‐KD and Hep3B‐KD cells and their respective control cells. (B) GO and KEGG analyses of DEGs in Huh7‐KD and Hep3B‐KD cells identified potential signaling pathways regulated by ADISSP in HCC. (C) WB analysis confirmed the effects of ADISSP expression on the NF‐κB signaling pathway in HCC. (D) Nuclear/cytoplasmic protein fractionation revealed that shADISSP increased cytoplasmic levels of phosphorylated p65. (E) IF staining results showing the change in p65. Scale bars, 20 µm. (F) WB analysis revealed that TNF‐α treatment increased p‐IκBα and p‐p65 levels in Huh7‐KD and Hep3B‐KD cells to some extent. (G) WB analysis revealed changes after p65 was knocked down in ADISSP‐OE cells. (H) WB analysis was used to assess the change of SLC16A3 and CCL20 expressions. (I) ELISA was used to assess the change of CCL20 level. Mean ± SD, *n* = 3, Student's *t*‐test, ^**^ *p* < 0.01, ^***^ *p* < 0.001. (J) Binding sites of RELA to the CCL20 and SLC16A3 promoters, as predicted by the JASPAR database. (K) ChIP‐qPCR analysis demonstrated significant enrichment of RELA at the SLC16A3 and CCL20 promoters in HCC cells. Mean ± SD, *n* = 3, Student's *t*‐test, ^**^ *p* < 0.01, ^***^ *p* < 0.001. (L,M) Representative IHC staining of ADISSP with SLC16A3 and CCL20 in the HCC tissue microarray comprising HBV‐related HCC patients. Scale bar, 50 µm.

We next sought to identify the transcriptional effectors downstream of NF‐κB that may mediate the biological effects of ADISSP. By integrating the shared DEGs from Hep3B‐KD and Huh7‐KD cells with publicly available p65 ChIP‐seq datasets [26], we identified seven candidate target genes (Figure S3G). Among these candidates, SLC16A3, a key lactate transporter involved in metabolic adaptation, and CCL20, a chemokine that regulates monocyte/macrophage recruitment, were prioritized for further investigation based on their potential relevance to distinct downstream biological processes. We then examined whether SLC16A3 and CCL20 were directly regulated by ADISSP–NF‐κB signaling. First, qRT‐PCR and western blot (WB) analysis showed that ADISSP could positively regulate the mRNA and protein levels of SLC16A3 and CCL20 (Figure 2H and Figure S3H). Second, immunohistochemical analysis of subcutaneous tumor tissues showed that ADISSP depletion was accompanied by reduced SLC16A3 and CCL20 expression in vivo (Figure S3I). In addition, ELISA analysis of conditioned media demonstrated that CCL20 secretion was decreased following ADISSP depletion and increased following ADISSP overexpression (Figure 2I).

To determine whether the two genes were directly regulated by NF‐κB, we next analyzed their promoter regions and identified putative RELA/p65‐binding sites within both the SLC16A3 and CCL20 promoters (Figure 2J). ChIP‐qPCR demonstrated the significant enrichment of RELA at the predicted promoter regions of both genes (Figure 2K), supporting their direct transcriptional regulation by NF‐κB. More importantly, analysis of our HCC tissue microarray demonstrated significant positive correlations between ADISSP expression and the expression of SLC16A3 and CCL20 (Figure 2L,M and Figure S3J). Furthermore, patients with concurrent high expression of ADISSP and SLC16A3, as well as those with concurrent high expression of ADISSP and CCL20, exhibited worse OS and DFS than the corresponding low‐expression groups (Figure S3K). These findings support the clinical relevance of SLC16A3 and CCL20 as downstream effectors of ADISSP/NF‐κB signaling in HCC.

### 2.3 SLC16A3 Mediates ADISSP‐Dependent Metabolic Reprogramming in HCC Cells

SLC16A3 encodes the lactate transporter MCT4, which facilitates lactate export and contributes to glycolytic metabolic adaptation in cancer cells [27]. We therefore investigated whether SLC16A3 mediates the metabolic effects of ADISSP in HCC. To validate the transcriptomic enrichment findings, we first examined whether ADISSP regulates metabolic remodeling in HCC cells. Quantitative metabolic analyses demonstrated that ADISSP knockdown markedly altered cellular metabolic profiles, as evidenced by reduced extracellular lactate secretion accompanied by increased intracellular lactate retention, whereas ADISSP overexpression exerted the opposite effects (Figure 3A). ADISSP depletion showed reduced intracellular pyruvate and ATP production, whereas ADISSP overexpression restored these metabolic parameters (Figure 3B). Extracellular acidification rate (ECAR) analysis showed that ADISSP depletion significantly reduced glycolysis, glycolytic capacity, and glycolytic reserve, whereas ADISSP overexpression markedly enhanced these glycolytic parameters. Oxygen consumption rate (OCR) analysis demonstrated that ADISSP depletion significantly increased basal respiration, ATP‐linked respiration, maximal respiration, and spare respiratory capacity, whereas ADISSP overexpression produced the opposite phenotype. (Figure 3C,D and Figure S4A,B). These findings demonstrate that ADISSP depletion induces profound metabolic remodeling in HCC cells, characterized by impaired glycolysis and enhanced mitochondrial oxidative phosphorylation.

**FIGURE 3:**
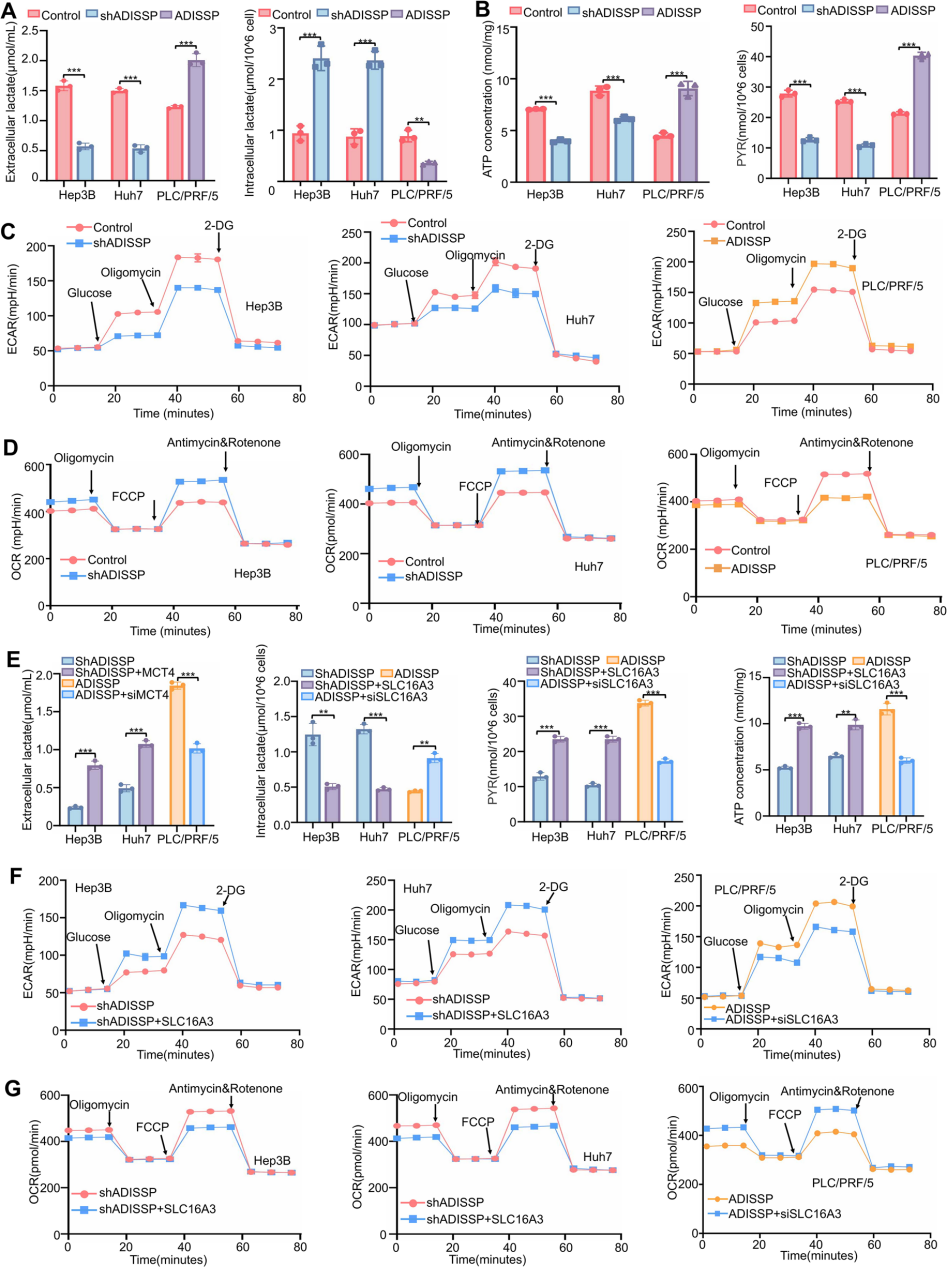
SLC16A3 mediates ADISSP‐dependent metabolic reprogramming in HCC cells. (A,B) Quantification of extracellular lactate, intracellular lactate, ATP, and pyruvate levels following ADISSP manipulation in HCC cells. Mean ± SD, *n* = 3, Student's *t*‐test, ^**^ *p* < 0.01, ^***^ *p* < 0.001. (C) ECAR profiles of Hep3B, Huh7, and PLC/PRF/5 cells were measured using the Seahorse assay. (D) OCR profiles of Hep3B, Huh7, and PLC/PRF/5 cells were measured using the Seahorse assay. (E) Extracellular lactate, intracellular lactate, pyruvate, and ATP levels were measured following SLC16A3 overexpression in ADISSP‐depleted cells and SLC16A3 knockdown in ADISSP‐OE cells. Mean ± SD, *n* = 3, Student's *t*‐test, ^**^ *p* < 0.01, ^***^ *p* < 0.001. (F) ECAR profiles in ADISSP‐KD HCC cells with SLC16A3 re‐expression and in ADISSP‐OE cells with SLC16A3 knockdown. (G) OCR profiles in ADISSP‐KD HCC cells with SLC16A3 re‐expression and in ADISSP‐OE cells with SLC16A3 knockdown.

As SLC16A3 has been identified as a direct downstream target of ADISSP/NF‐κB signaling, we next investigated whether SLC16A3 mediates the oncogenic functions of ADISSP. To determine whether SLC16A3 functionally mediates the effects of ADISSP, we performed reciprocal genetic rescue experiments. Ectopic overexpression of SLC16A3 in ADISSP‐KD HCC cells markedly restored the impaired proliferative, migratory, and invasive capacities caused by ADISSP depletion. Conversely, SLC16A3 knockdown in PLC/PRF/5‐OE cells significantly attenuated the enhanced proliferation, migration, and invasion induced by ADISSP overexpression (Figure S4C–G). More importantly, SLC16A3 overexpression in ADISSP‐KD HCC cells restored lactate distribution by increasing extracellular lactate release while reducing intracellular lactate accumulation, together with corresponding changes in ATP and pyruvate levels. Conversely, SLC16A3 depletion in PLC/PRF/5‐OE cells produced the opposite metabolic effects (Figure 3E). ECAR and OCR analyses further showed that SLC16A3 restoration recovered glycolytic activity and reduced mitochondrial respiration in ADISSP‐KD cells, whereas SLC16A3 depletion reversed these effects in ADISSP‐overexpressing cells (Figure 3F,G and Figure S4H,I). These results establish SLC16A3 as a key mediator of ADISSP‐dependent metabolic reprogramming in HCC cells.

### 2.4 SLC16A3 Mediates ADISSP‐Induced Ferroptosis Resistance in HCC

Given that metabolic reprogramming can influence cellular redox homeostasis and ferroptosis susceptibility [28], we examined whether ADISSP depletion alters basal oxidative stress. Compared with control cells, ADISSP‐depleted HCC cells exhibited significantly increased intracellular ROS accumulation under basal conditions (Figure 4A and Figure S5A). Then, we attempt to investigate whether ADISSP regulates ferroptosis susceptibility in HCC. First, we analyzed the expression correlation of ADISSP and several ferroptosis‐related genes such as ACSL4, FTH1, NCOA4, and GPX4 in HCC tissues. We found that ADISSP expression was positively correlated with GPX4 expression in HBV‐related HCC tissues (Figure S5B). To determine whether ADISSP depletion enhances ferroptotic susceptibility, we treated HCC cells with 20 µM erastin. ADISSP‐depleted HCC cells exhibited markedly increased lipid peroxidation following erastin treatment, whereas cotreatment with 10 µM ferrostatin‐1 (Fer‐1) substantially reversed this effect (Figure 4B and Figure S5C,D). Consistently, erastin stimulation resulted in increased malondialdehyde (MDA) accumulation and intracellular Fe^2^^+^ levels in ADISSP‐depleted cells (Figure 4C,D and Figure S5E,F). Moreover, erastin exerted greater growth‐inhibitory and cytotoxic effects in ADISSP‐depleted cells than in control cells, whereas Fer‐1 co‐treatment markedly attenuated these effects (Figure 4E and Figure S5G,H). In parallel, we confirmed that ADISSP depletion markedly reduced GPX4 expression in HCC in vitro. IHC analysis of subcutaneous tumor tissues demonstrated decreased GPX4 expression together with increased 4‐hydroxynonenal (4‐HNE) staining in vivo (Figure 4F,G). Consistently, treatment with 0.5 µM RSL3 markedly increased lipid peroxidation in ADISSP‐depleted cells, whereas co‐treatment with 0.5 µm liproxstatin‐1 (Lip‐1) substantially attenuated this increase (Figure 4H and Figure S5I). RSL3‐induced cell death in ADISSP‐depleted cells was markedly reversed by 0.5 µm Lip‐1, whereas the apoptosis inhibitor Z‐VAD‐FMK (20 µm) and the necroptosis inhibitor Nec‐1s (10 µm) failed to provide comparable protection (Figure 4I and Figure S5J). These findings demonstrate that ADISSP depletion renders HCC cells more susceptible to ferroptotic stress.

**FIGURE 4:**
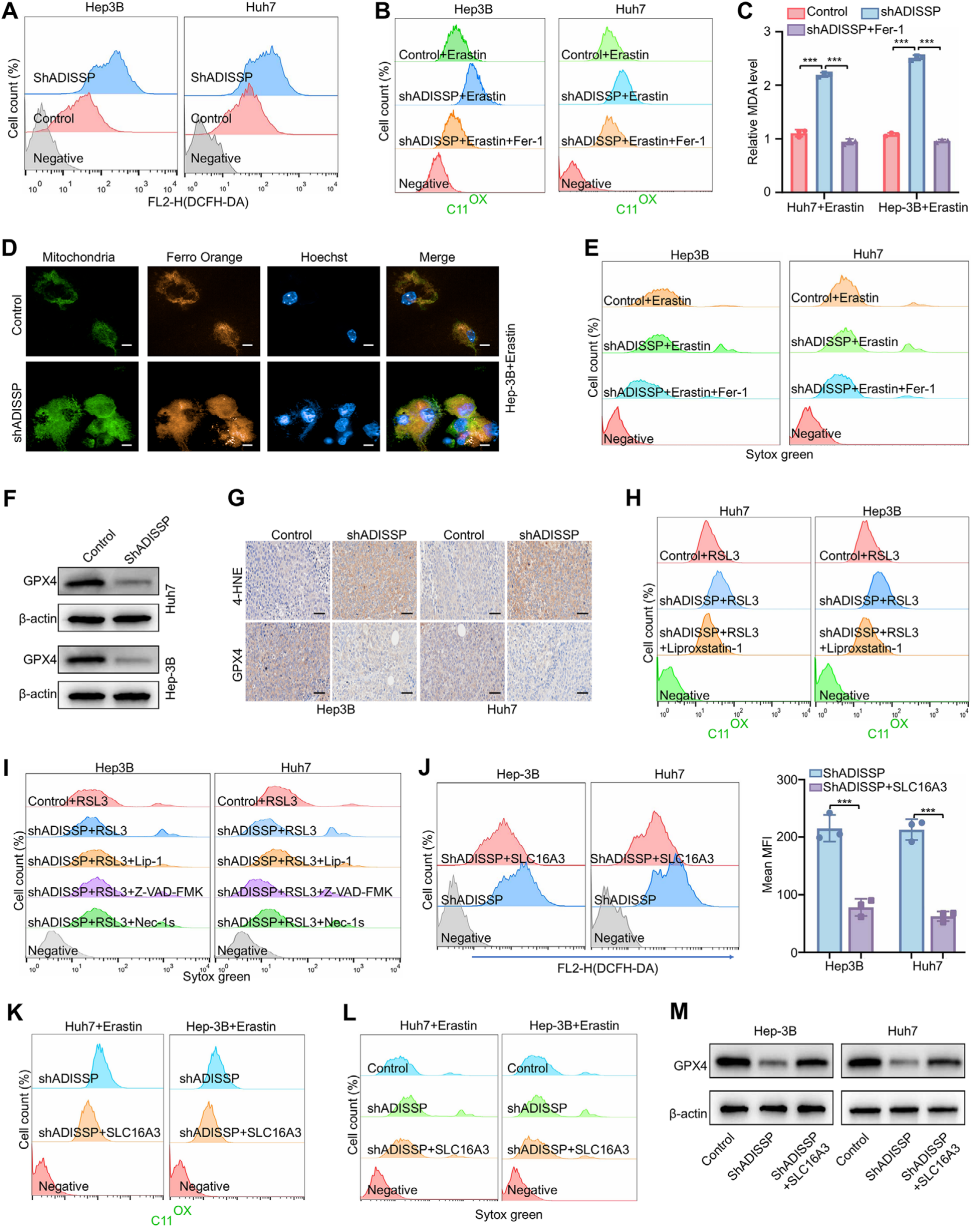
ADISSP promotes ferroptosis resistance through SLC16A3 in HCC cells. (A) Flow cytometric analysis of intracellular ROS levels in control and ADISSP‐depleted HCC cells. (B) Lipid peroxidation was assessed by flow cytometric analysis of BODIPY 581/591 C11‐stained cells following treatment with erastin (20 µM) with or without ferrostatin‐1 (Fer‐1, 10 µm). (C) MDA levels in HCC cells exposed to erastin or/and Fer‐1. Mean ± SD, *n* = 3, Student's *t*‐test, ^***^ *p* < 0.001. (D) FerroOrange and MitoTracker costaining demonstrated enhanced Fe^2+^ accumulation in shADISSP cells following erastin treatment. Scale bars, 10 µm. (E) Cell death under the indicated conditions was assessed by flow cytometry using SYTOX Green staining. (F) WB analysis of GPX4 protein levels in shADISSP cells. (G) IHC analysis of GPX4 and 4‐HNE expression in tumor tissues. Scale bars, 50 µm. (H) Lipid peroxidation was assessed by flow cytometric analysis of BODIPY 581/591 C11‐stained cells following treatment with RSL3 with or without liproxstatin‐1 (Lip‐1, 0.5 µm). (I) Cell death was assessed by SYTOX Green staining and flow cytometry following treatment with 0.5 µm RSL3 alone or in combination with 0.5 µm Lip‐1, the apoptosis inhibitor Z‐VAD‐FMK (20 µm), or the necroptosis inhibitor Nec‐1s (10 µm). (J) Flow cytometry was used to detect changes in ROS. Mean ± SD, *n* = 3, Student's *t*‐test, ^***^ *p* < 0.001. (K) Lipid peroxidation was assessed by flow cytometric analysis of BODIPY 581/591 C11‐stained cells following treatment with 20 µm erastin. (L) Erastin‐induced cell death was assessed by SYTOX Green staining and flow cytometry following treatment with 20 µm erastin. (M) WB analysis of GPX4 protein levels in HCC cells.

We next investigated whether SLC16A3 also accounts for the ferroptosis‐regulation maintained by ADISSP. Restoration of SLC16A3 expression significantly reduced the elevated intracellular ROS levels induced by ADISSP depletion (Figure 4J). Consistently, SLC16A3 overexpression markedly attenuated erastin‐induced lipid peroxidation and decreased MDA accumulation in ADISSP‐deficient cells (Figure 4K and Figure S5K,L). Furthermore, SLC16A3 restoration significantly alleviated erastin‐induced cell death in ADISSP‐depleted HCC cells (Figure 4L and Figure S5M). WB analysis further demonstrated that SLC16A3 overexpression restored GPX4 expression in ADISSP‐depleted cells (Figure 4M). These findings demonstrate that SLC16A3 is a critical downstream mediator through which ADISSP regulates metabolic adaptation and ferroptosis sensitivity in HCC cells.

### 2.5 ADISSP Promotes Immunosuppressive Remodeling of the HCC Tumor Microenvironment

As transcriptomic analysis revealed significant enrichment of immune‐related pathways, we further performed immune infiltration analysis, which demonstrated a strong positive association between ADISSP expression and macrophage infiltration (Figure S6A). These findings prompted us to investigate whether ADISSP regulates macrophage polarization and recruitment. THP‐1 cells were cultured and differentiated into macrophages using phorbol‐12‐myristate‐13‐acetate (PMA) and subsequently co‐cultured with HCC cells (Figure 5A). Quantitative PCR analysis demonstrated that the expression of M2‐associated markers, including CD206, ARG1, IL10, and TGFβ1, was markedly decreased in macrophages co‐cultured with ADISSP‐depleted HCC cells, whereas ADISSP overexpression significantly increased the expression of these markers (Figure 5B and Figure S6B). Consistently, ELISA revealed that the secretion of the immunosuppressive cytokines IL10 and TGFβ1 was significantly reduced following ADISSP depletion but markedly elevated upon ADISSP overexpression (Figure S6C,D). Flow cytometric analysis further confirmed that ADISSP knockdown significantly reduced the proportion of CD206‐positive macrophages, whereas ADISSP overexpression markedly promoted M2‐like macrophage polarization (Figure 5C,D). Moreover, we isolated the bone marrow‐derived macrophages (BMDMs) of mouse. Then, BMDMs were co‐cultured with control or shADISSP Hepa1‐6 cells. Consistently, TAMs from the shADISSP group exhibited decreased CD206 and increased iNOS expression by flow cytometry, accompanied by reduced CD206 and ARG1 and increased CD86 and iNOS expression at the mRNA level, indicating attenuated M2‐like polarization (Figure 5E,F and Figure S6E). To evaluate the immunomodulatory effects of shADISSP on CD8(+) T cells, BMDMs were then co‐cultured with mouse CD8(+) T cells isolated from the spleen tissue of C57BL/6 mice. We found that the CD8(+) T cells of the shADISSP group possessed better growth vitality than the control group (Figure 5G and Figure S6F). To further validate these findings in vivo, ADISSP was stably silenced in Hepa1‐6 cells before subcutaneous implantation (Figure S6G). Flow cytometric and immunofluorescence analyses consistently demonstrated that ADISSP depletion markedly reduced the infiltration of M2‐like macrophages within the tumor microenvironment (Figure 5H,I).

**FIGURE 5:**
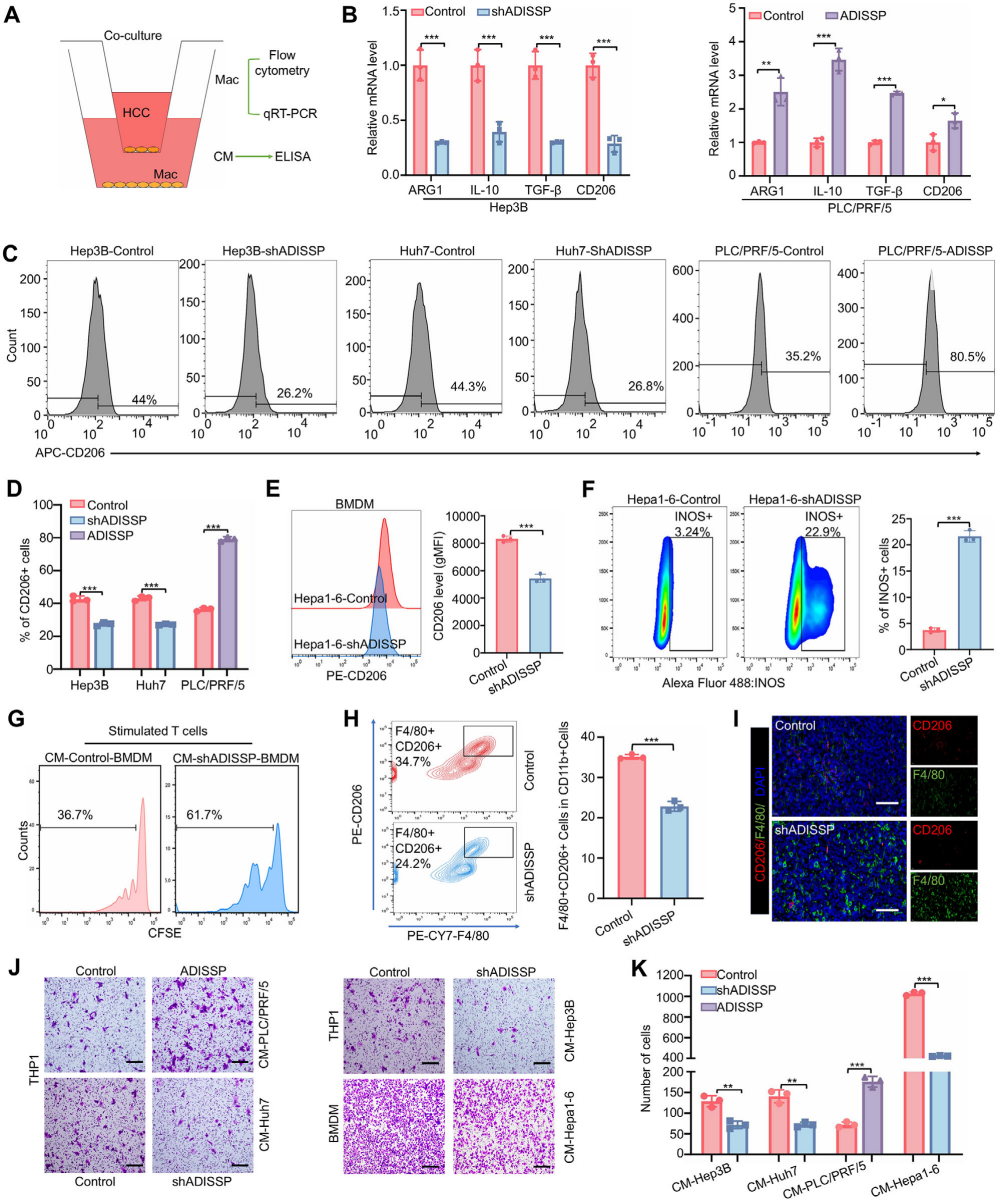
ADISSP promotes immunosuppressive macrophage remodeling in HCC. (A) Schematic diagram showing HCC cells cocultured with macrophages using a 0.4 µm pore size Transwell system. (B) CD206, ARG1, IL‐10, and TGF‐β1 mRNA levels in THP‐1‐derived macrophages were measured by qRT‒PCR. Mean ± SD, *n* = 3, Student's *t*‐test, ^*^ *p* < 0.05, ^**^ *p* < 0.01, ^***^ *p* < 0.001. (C, D) Flow cytometric analysis of CD206 expression in THP‐1‐derived macrophages. Mean ± SD, *n* = 3, Student's *t*‐test, ^***^ *p* < 0.001. (E, F) Flow cytometric analysis of CD206 (E) and iNOS (F) expression within the CD45^+^CD11b^+^F4/80^+^ BMDM population. Mean ± SD, *n* = 3, Student's t‐test, ^***^ *p* < 0.001. (G) CFSE‐based flow cytometric analysis of CD8(+) T cells proliferation following co‐culture with BMDMs under the indicated conditions. (H) Flow cytometry analysis revealed a decrease in the infiltration of CD206(+) macrophages. mean ± SD, *n* = 3, Student's *t*‐test, ^***^ *p* < 0.001. (I) Immunofluorescence analyses revealed a decrease in CD206(+) cells. Scale bars, 20 µm. (J,K) Transwell recruitment assays of THP‐1‐derived macrophages and primary BMDMs using conditioned media from the indicated HCC cells as chemoattractants. Scale bars, 100 µm. mean ± SD, *n* = 3, Student's *t*‐test, ^**^ *p* < 0.01, ^***^ *p* < 0.001.

We subsequently assessed whether ADISSP also influences macrophage recruitment. Chemotactic migration assays demonstrated that conditioned medium derived from ADISSP‐KD HCC cells significantly impaired macrophage migration, whereas conditioned medium from ADISSP‐OE cells markedly enhanced macrophage recruitment (Figure 5J,K). These findings suggest that ADISSP promotes immunosuppressive macrophage remodeling in HCC, characterized by enhanced macrophage recruitment, increased M2‐like polarization, and impaired CD8(+) T cell activity.

### 2.6 SLC16A3‐Dependent Lactate Signaling and CCL20‐Mediated Macrophage Recruitment Cooperatively Establish an Immunosuppressive Tumor Microenvironment

Given the pivotal role of STAT3 signaling in the biology function of ADISSP in HCC, we next examined STAT3 activation in THP‐1‐derived macrophages. WB analysis revealed that conditioned medium from ADISSP‐depleted HCC cells markedly reduced STAT3 phosphorylation, whereas conditioned medium from ADISSP‐OE cells significantly enhanced STAT3 activation (Figure 6A). We next examined the role of SLC16A3 in macrophage polarization. SLC16A3 overexpression restored the impaired M2‐like polarization induced by ADISSP depletion, whereas SLC16A3 knockdown attenuated the enhanced M2‐like polarization driven by ADISSP overexpression (Figure 6B–D and Figure S7A–C). Then, we investigated whether lactate contributes to this effect. Supplementation with 5 mm lactic acid markedly restored the reduced M2‐like polarization of macrophages exposed to conditioned medium from ADISSP‐depleted HCC cells (Figure S7D–H). Importantly, exogenous lactate restored the impaired STAT3 phosphorylation induced by ADISSP depletion, while SLC16A3 knockdown abolished STAT3 activation elicited by ADISSP overexpression (Figure S7I). Because lactic acid supplementation simultaneously alters extracellular lactate levels and pH, we performed additional experiments to distinguish the contribution of lactate from that of extracellular acidification. Sodium lactate was supplemented at a concentration comparable to that measured in conditioned medium from ADISSP‐overexpressing HCC cells. In macrophages exposed to conditioned medium from ADISSP‐OE cells with SLC16A3 depletion, supplementation with 2 mm sodium lactate substantially restored STAT3 phosphorylation (Figure 6E). We next performed pH‐matched experiments to dissect the respective contributions of lactate accumulation and extracellular acidification. Both sodium lactate under neutral conditions and extracellular acidification in the absence of added lactate enhanced STAT3 phosphorylation and M2‐like macrophage polarization (Figure S7J,K). These results indicate that both lactate accumulation and extracellular acidification contribute to macrophage STAT3 activation.

**FIGURE 6:**
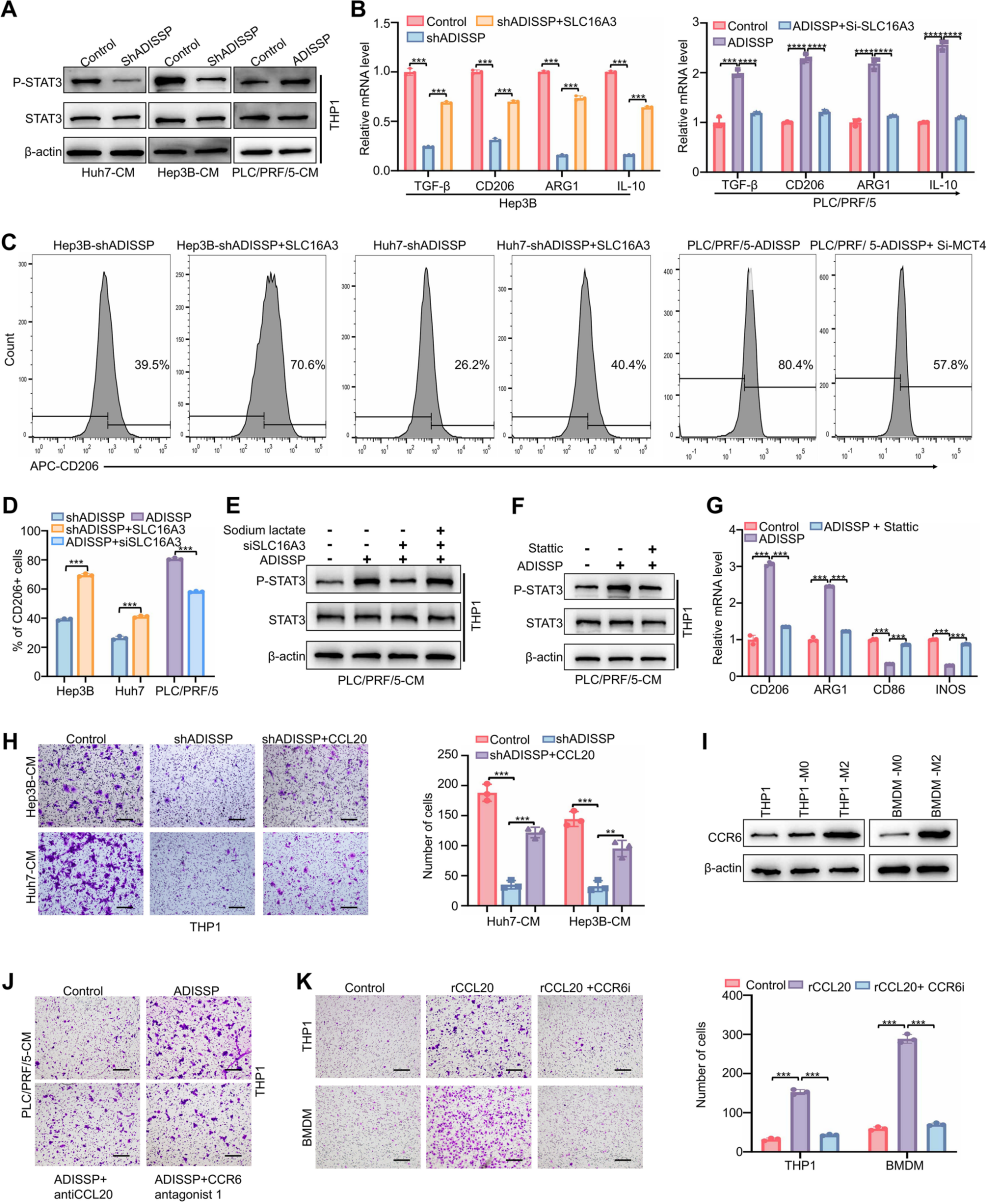
SLC16A3–lactate–STAT3 and CCL20–CCR6 signaling mediate ADISSP‐driven macrophage polarization and recruitment. (A) Representative western blot analysis of p‐STAT3 and STAT3 in THP‐1‐derived macrophages cultured with CM from control, sh‐ADISSP, or ADISSP‐OE HCC cells. (B) CD206, ARG1, IL‐10, and TGF‐β mRNA levels in THP‐1‐derived macrophages were measured by qRT‒PCR. mean ± SD, *n* = 3, Student's *t*‐test, ^***^ *p* < 0.001, ^****^ *p* < 0.0001. (C, D) Quantification of CD206 expression in macrophages using FCM analysis. Mean ± SD, *n* = 3, Student's *t*‐test, ^***^ *p* < 0.001. (E) WB analysis of p‐STAT3 and total STAT3 in THP‐1‐derived macrophages cultured with conditioned medium from ADISSP‐OE HCC cells with SLC16A3 knockdown, with or without 2 mm sodium lactate supplementation. (F) Representative WB analysis of p‐STAT3 and total STAT3 in THP‐1‐derived macrophages exposed to conditioned medium from ADISSP‐OE HCC cells, with or without Stattic (2.5 µm) treatment. (G) CD206, ARG1, CD86, and iNOS mRNA levels in primary BMDMs were measured by qRT‒PCR. Mean ± SD, *n* = 3, Student's *t*‐test, ^***^ *p* < 0.001. (H) Chemotactic migration assays of macrophages co‐cultured with HCC cells. Scale bars, 100 µm. Mean ± SD, *n* = 3, Student's *t*‐test, ^**^ *p* < 0.01, ^***^ *p* < 0.001. (I) WB analysis of CCR6 protein expression in THP‐1‐derived macrophages and primary BMDMs. (J) Transwell recruitment assays of THP‐1‐derived macrophages using conditioned medium from ADISSP‐OE HCC cells, with or without an anti‐CCL20 neutralizing antibody or CCR6 antagonist 1. Scale bars, 100 µm. (K) Transwell recruitment assays of THP‐1‐derived macrophages and primary BMDMs in response to recombinant CCL20 (rCCL20), with or without CCR6 antagonist 1. Scale bars, 100 µm. Mean ± SD, *n* = 3, Student's *t*‐test, ^***^ *p* < 0.001.

To further determine whether STAT3 activation is functionally required for ADISSP‐induced macrophage polarization, we treated macrophages exposed to conditioned medium from ADISSP‐OE HCC cells with the STAT3 inhibitor Stattic. Stattic (2.5 µM) markedly attenuated ADISSP‐induced STAT3 phosphorylation and suppressed M2‐like macrophage polarization, as evidenced by reduced expression of M2‐associated markers and restoration of M1‐associated markers (Figure 6F,G). These findings support a model in which ADISSP‐driven SLC16A3 upregulation promotes a lactate‐rich and acidic extracellular milieu, with both lactate accumulation and extracellular acidification contributing to STAT3 activation and subsequent M2‐like macrophage polarization.

In parallel, we investigated whether CCL20 mediates macrophage recruitment. Restoration of CCL20 expression significantly rescued the impaired macrophage migration caused by ADISSP depletion (Figure 6H). CCL20 is classically associated with recruitment of CCR6‐positive cells. To determine whether CCR6 mediated CCL20‐induced macrophage M2 polarization and recruitment [29], we detected the change in CCR6 expression during macrophage M2 polarization. A higher expression of CCR6 was detected in M2‐polarized macrophages than in M0 macrophages in both THP‐1‐derived macrophages and primary BMDMs (Figure 6I). Then, we detected the influence of the CCL20‒CCR6 axis in macrophage recruitment. Our results showed that recombinant CCL20 directly promoted macrophage migration in vitro. Moreover, both CCL20‐neutralizing antibody and pharmacological inhibition of CCR6 markedly attenuated ADISSP‐OE‐induced macrophage migration [30] (Figure 6J and Figure S7L). To further establish the functional role of CCR6 in CCL20‐mediated chemotaxis, we examined the response to recombinant CCL20 in both THP‐1‐derived macrophages and primary BMDMs. CCR6 blockade significantly suppressed recombinant CCL20‐induced migration in both macrophage models (Figure 6K). These findings identify the CCL20–CCR6 axis as a key mediator of macrophage recruitment downstream of ADISSP.

### 2.7 ADISSP Activates NF‐κB Signaling by Suppressing PPP1CA‐Mediated IKKβ Dephosphorylation

We next sought to determine how ADISSP mechanistically activates NF‐κB pathway. Protein–protein interaction analysis using the STRING database identified PPP1CA as a potential ADISSP‐interacting protein [31] (Figure 7A). As PPP1CA is a serine/threonine phosphatase known to regulate phosphorylation‐dependent signaling, we hypothesized that ADISSP activates NF‐κB through PPP1CA. Co‐immunoprecipitation and immunofluorescence assays confirmed a robust interaction between ADISSP and PPP1CA (Figure 7B,C). Notably, PPP1CA also interacted with IKKβ, the upstream kinase responsible for NF‐κB activation (Figure 7D and Figure S8A), suggesting that ADISSP may regulate NF‐κB signaling by modulating PPP1CA‐mediated dephosphorylation of IKKβ. To test this possibility, ADISSP and PPP1CA were simultaneously manipulated in HCC cells. PPP1CA depletion largely abolished the inhibitory effect of ADISSP knockdown on IKKβ phosphorylation, whereas ectopic expression of PPP1CA restored the phosphorylation changes induced by ADISSP overexpression (Figure 7E,F). These findings demonstrate that ADISSP regulates IKKβ phosphorylation in a PPP1CA‐dependent manner.

**FIGURE 7:**
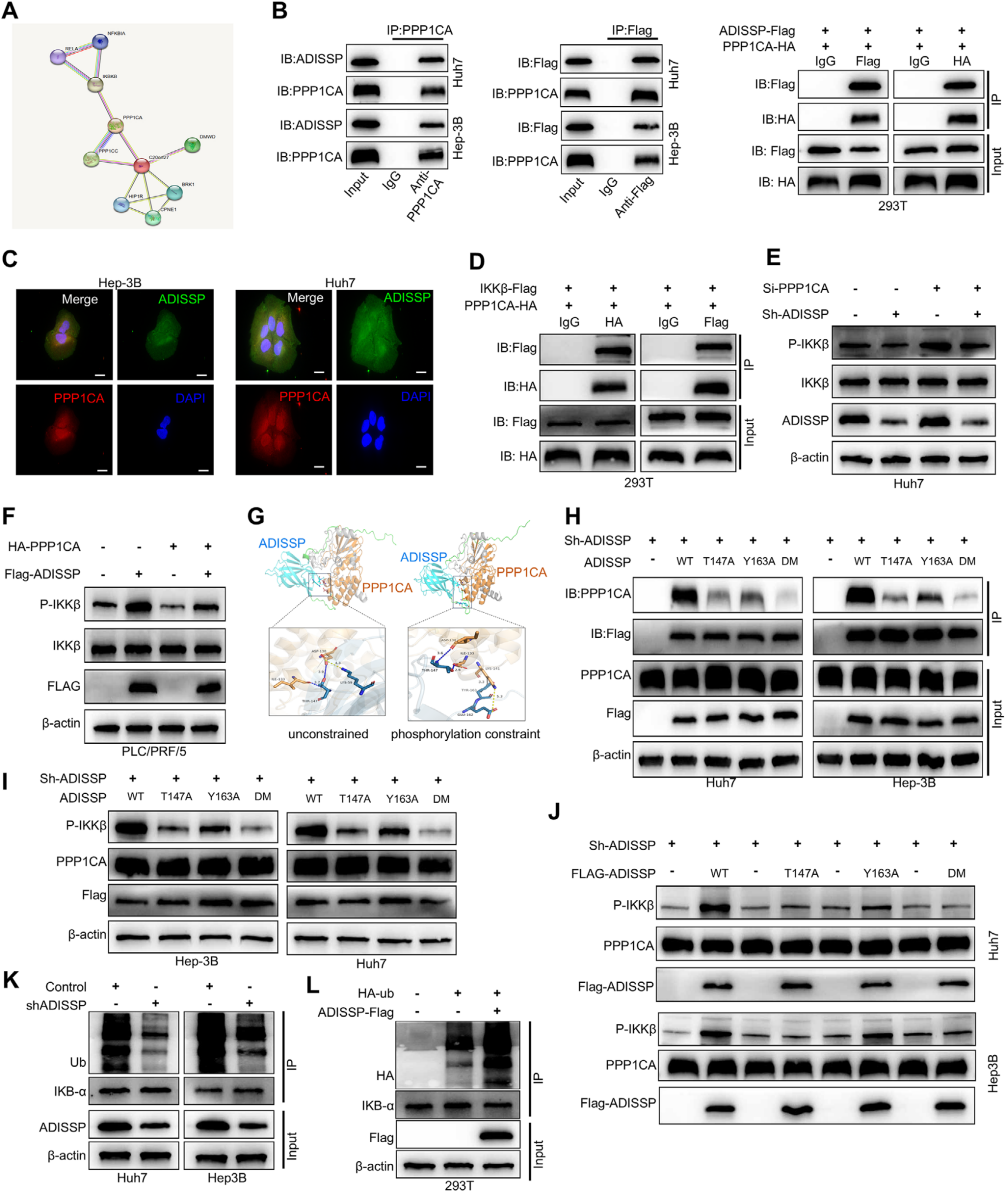
ADISSP sustains NF‐κB activation through inhibition of PPP1CA‐mediated IKKβ dephosphorylation. (A) Predicted interacting proteins of ADISSP based on the STRING database. (B, C) Co‐IP and IF assays demonstrated that ADISSP could interact with PPP1CA directly. (D) Co‐IP assays demonstrated that PPP1CA could interact with IKKβ directly. (E) WB analysis of P‐IKKβ levels in ADISSP‐KD HCC cells transfected with si‐PPP1CA (F) WB analysis of P‐IKKβ levels in ADISSP‐OE HCC cells transfected with PPP1CA plasmids. (G) 3D map of the predicted interaction sites between ADISSP and PPP1CA. (H) Effect of ADISSP mutants on ADISSP–PPP1CA binding. (I) Changes in IKKβ phosphorylation induced by different ADISSP mutants. (J) In vitro dephosphorylation assays revealed that different ADISSP mutants differentially altered their inhibitory effects on PPP1CA‐mediated dephosphorylation of IKKβ at Ser176/180. (K) WB analysis revealed that shADISSP reduced the ubiquitination level of IKBα. (L) WB analysis revealed that ADISSP overexpression increased IKBα ubiquitination.

To elucidate how ADISSP regulates PPP1CA activity, structural modeling was performed to identify potential binding interfaces. The predicted model suggested that Thr147, together with Tyr163 and Glu162, forms a critical interface mediating the interaction between ADISSP and the catalytic region of PPP1CA, potentially restricting substrate access to its catalytic pocket (Figure 7G). To experimentally validate this prediction, ADISSP mutants (T147A, Y163A, and T147A/Y163A) were generated. Co‐immunoprecipitation assays demonstrated that mutation of Thr147 markedly weakened the interaction between ADISSP and PPP1CA, whereas Y163A moderately reduced binding. Consistently, the double mutant almost completely abolished this interaction (Figure 7H). Disruption of the ADISSP‐PPP1CA interaction markedly attenuated IKKβ phosphorylation (Figure 7I). In vitro phosphatase assays showed that PPP1CA efficiently dephosphorylated IKKβ, whereas wild‐type ADISSP significantly inhibited this phosphatase activity. In contrast, ADISSP mutants largely lost their ability to suppress PPP1CA‐mediated dephosphorylation (Figure 7J). More important, re‐expression of wild‐type ADISSP, but not the T147A/Y163A double mutant, rescued the impaired growth, migration, and invasion of ADISSP‐KD HCC cells (Figure S8B,C). Since phosphorylation of IKKβ triggers IκBα ubiquitination and proteasomal degradation, we also examined whether the ADISSP‐PPP1CA axis regulates this downstream event. ADISSP overexpression markedly promoted IκBα ubiquitination and degradation, whereas ADISSP depletion produced the opposite effects (Figure 7K,L). These findings demonstrate that ADISSP suppresses PPP1CA phosphatase activity to sustain IKKβ phosphorylation, thereby facilitating IκBα degradation, activating NF‐κB signaling, and ultimately promoting HCC progression.

### 2.8 Targeting ADISSP Remodels the Tumor Immune Microenvironment and Enhances the Therapeutic Efficacy of Combined Anti‐PD‐1 and Lenvatinib Treatment

Emerging evidence has demonstrated that targeting aerobic glycolysis can effectively overcome lenvatinib resistance in HCC [32, 33] and that TAMs could mediate the resistance to ICB therapy [34, 35]. As ADISSP was involved in regulating tumour metabolic reprogramming and macrophage polarization in HCC, we further attempted to determine whether targeting ADISSP could enhance the efficacy of combination therapy with anti‐PD1 therapy and lenvatinib in HCC. First, the control and sh‐ADISSP Hepa1‐6 cells were subcutaneously inoculated into C57BL/6 mice, and were subsequently treated with anti‐PD1 therapy combined with lenvatinib (Figure 8A). We found that ADISSP‐KD markedly enhanced the tumour growth inhibition induced by lenvatinib and anti‐PD1 treatment (Figure 8B,C). To determine whether the enhanced therapeutic response was associated with remodeling of the tumor immune microenvironment, immunohistochemical staining was performed on tumor tissues. ADISSP depletion combined with anti‐PD‐1 and lenvatinib treatment markedly reduced macrophage infiltration, particularly M2 macrophages, while increasing CD8(+) T cells infiltration. In contrast, CD4(+) T cells infiltration was reduced in the combination‐treated sh‐ADISSP tumors (Figure 8D). Flow cytometric analysis also demonstrated that tumors from the sh‐ADISSP mice receiving combined anti‐PD‐1 and lenvatinib treatment exhibited significantly reduced proportions of CD206(+) macrophages, together with increased proportions of iNOS(+) macrophages and CD8(+) T cells (Figure 8E and Figure S9A–C). Multiplex immunofluorescence demonstrated a marked reduction in CD206(+) macrophages as well as increased infiltration of CD86(+) macrophages and Granzyme B (+) and CD8(+) cytotoxic T cells in the sh‐ADISSP combination group (Figure 8F). These findings demonstrate that ADISSP depletion markedly enhances the therapeutic efficacy of combined anti‐PD‐1 and lenvatinib treatment by reprogramming the tumor immune microenvironment toward an antitumor state characterized by reduced M2 macrophage polarization and enhanced cytotoxic CD8(+) T cells immunity.

**FIGURE 8:**
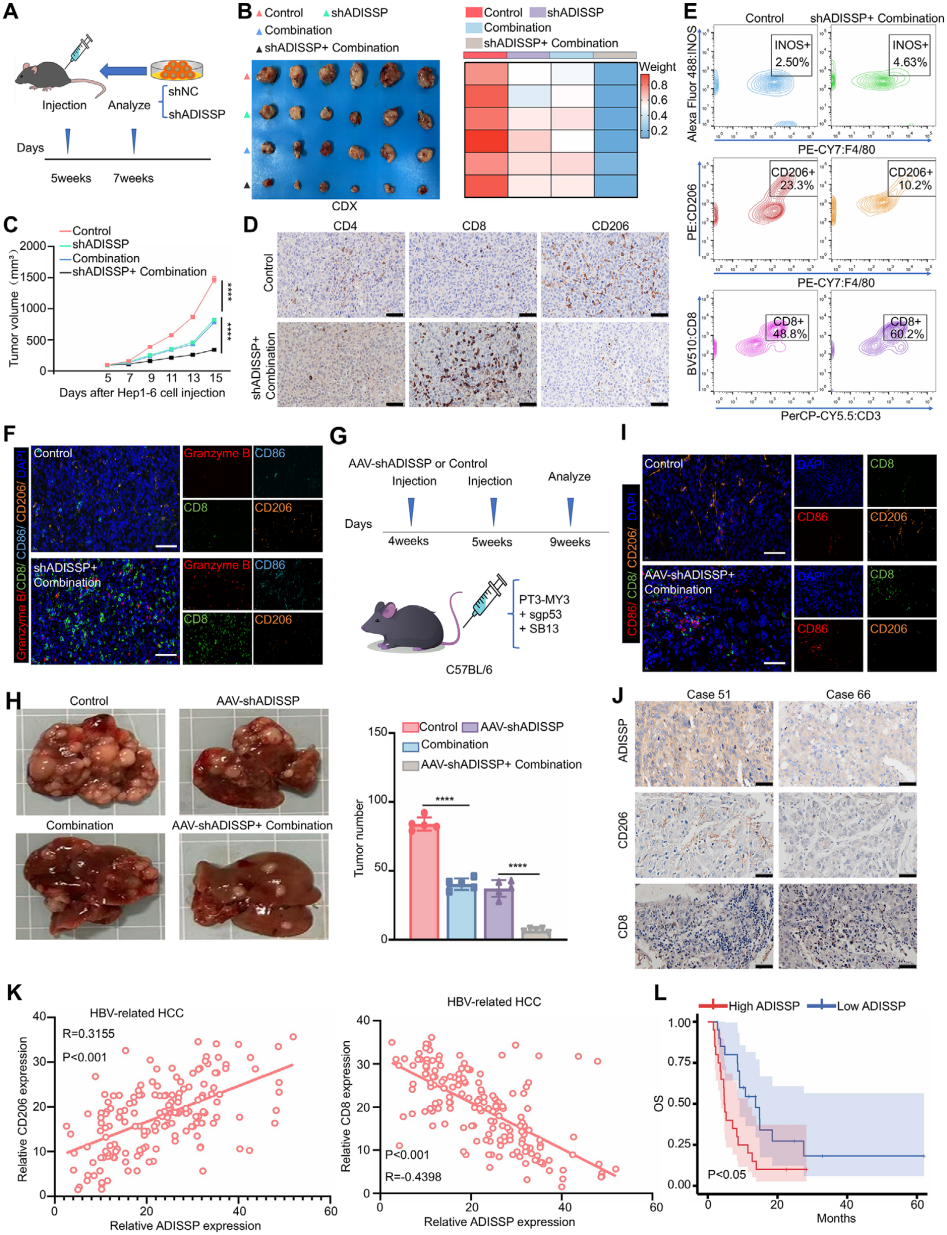
Targeting ADISSP potentiates anti‐PD‐1 plus lenvatinib therapy through immune microenvironment remodeling. (A) Schematic diagram. (B,C) Xenograft tumor model formed by the injection of Hepa1‐6 cells. The combination of anti‐PD‐1 and lenvatinib, together with ADISSP knockdown significantly suppressed tumor growth in xenograft models. Mean ± SD, *n* = 6, two‐way ANOVA, ^**^ *p* < 0.01, ^***^ *p* < 0.001, ^****^ *p* < 0.0001. (D) Immunohistochemistry analyses revealed a decrease in CD4(+) T cells and CD206+ cells, accompanied by an increase in CD8(+) T cells in shADISSP group. Scale bars, 50 µm. (E) Representative maps and percentages of tumor‐infiltrating iNOS(+) macrophages, CD206(+) macrophages, and CD8(+) T cells in HCC tumors of the control group and shADISSP with combination therapy group. (F) Immunofluorescence analyses revealed an increase in Granzyme (+) CD8(+) T cells as well as CD86(+) cells, accompanied by a decrease in CD206(+) cells. Scale bars, 20 µm. (G) Schematic representation of primary tumor formation. (H) Representative gross images of livers and quantitative analysis of tumor number in mice receiving vehicle, shADISSP, combination therapy, or the shADISSP with combination treatment (n = 5). Mean ± SD, *n* = 5, two‐way ANOVA, ^****^ *p* < 0.0001. (I) mIF staining of primary tumours with ADISSP inhibition in combination with the two drugs revealed the increased infiltration of CD86(+) cells and CD8(+) T cells and a significant reduction in CD206(+) cells infiltration. Scale bars, 20 µm. (J,K) Representative IHC images of ADISSP, CD206, and CD8 in serial TMAs of HBV‐positive HCC samples, together with correlation analyses between ADISSP and CD206 expression and between ADISSP and CD8 expression. Scale bars, 50 µm. (L) The Kaplan‐Meier survival analysis showed that high ADISSP expression was associated with poor prognosis in HBV‐related unresectable HCC patients treated by pembrolizumab plus lenvatinib. The clinical characteristics of 40 HBV‐related HCC patients were provided in Table S7.

To further validate the therapeutic effect of targeting ADISSP in combination with anti‐PD1 and lenvatinib treatment, we established primary HCC models in mice via plasmid injection (Figure 8G). Consistent with the findings in the subcutaneous tumour model, the combination therapy of ADISSP inhibition and anti‐PD1 plus lenvatinib treatment significantly suppressed tumour initiation and progression compared with the other treatments (Figure 8H). ADISSP inhibition in combination with anti‐PD‐1 and lenvatinib reduced M2 macrophage infiltration, increased CD86(+) macrophage and CD8(+) T cells infiltration (Figure 8I). Furthermore, ADISSP expression in human HCC specimens was positively correlated with increased CD206(+) macrophages infiltration and inversely correlated with CD8(+) T cells (Figure 8J,K). Finally, we retrospectively evaluated the effcacy of the anti‐PD1 inhibitor plus lenvatinib in 40 unresectable HBV‐related HCC (uHCC) patients with histologically confrmed received lenvatinib (body weight ≥ 60 kg, 12 mg; < 60 kg, 8 mg) orally daily and pembrolizumab 200 mg in advanced HCC by ultrasonography guided biopsy. Patients enrolled in our study intravenously on day 1 of a 21‐day cycle. Objective response rate (ORR) was assessed by RECIST version 1.1 per independent imaging review. The end points were ORR and DOR by investigator assessment and overall survival. Immunohistochemistry was used to detect the ADISSP expression in HCC tissue from the needle biopsy. Then, we analyzed retrospectively the relationship between ADISSP expression and the prognosis of unresectable HCC patients receiving anti‐PD‐1 plus lenvatinib treatment. The Kaplan–Meier survival analysis showed that high ADISSP expression was associated with poor prognosis in HBV‐related unresectable HCC patients (Figure 8L). Thus, we conclude that targeting ADISSP enhances the antitumour efficacy of anti‐PD‐1 plus lenvatinib treatment by reducing M2 macrophage infiltration and promoting CD8(+) T cell activation.

## 3 Discussion

In this study, we identify ADISSP as a previously unrecognized molecular hub that integrates tumor metabolic reprogramming with immune suppression in HBV‐related HCC. Although metabolic adaptation and immune evasion are well‐established determinants of HCC progression and therapeutic resistance [36], the upstream regulators coordinating these two biological processes remain largely unknown. Our findings demonstrate that ADISSP is markedly up‐regulated in HCC and functions as an oncogenic driver by simultaneously remodeling tumor cell metabolism and the immune microenvironment. Notably, ADISSP depletion reduced glycolytic activity but increased basal, ATP‐linked, and maximal respiration as well as spare respiratory capacity. Our findings in vitro and in vivo support a model in which ADISSP upregulation may regulate mitochondrial respiratory capacity and respiratory headroom to promote HCC progression. ADISSP also contributes to the establishment of an immunosuppressive tumor microenvironment in HBV‐related HCC. More important, ADISSP inhibition enhanced the efficacy of anti‐PD‐1 plus lenvatinib treatment in our preclinical models, supporting its potential therapeutic relevance. However, HCC tumors in clinical may naturally upregulates ADISSP, which likely impairs mitochondrial quality and respiratory headroom, thereby driving a compensatory switch that empowers glycolysis. For those patients with HCC of ADISSP high expression, targeting ADISSP with its inhibitor may achieve favorable therapeutic outcomes by disturbing the glycolysis process.

Mechanistically, we uncover a previously unrecognized mode of sustained NF‐κB activation mediated by ADISSP. Unlike canonical NF‐κB activation driven primarily by inflammatory cytokines or upstream kinase stimulation [37], ADISSP directly interacts with PPP1CA and suppresses PPP1CA‐mediated IKKβ dephosphorylation, thereby maintaining persistent NF‐κB signaling activity. This finding expands the current understanding of NF‐κB regulation by revealing that tumor‐associated proteins can modulate transcriptional signaling output through direct control of phosphatase activity. Given the central role of NF‐κB in regulating inflammation, metabolism, and immune responses [19, 38], the ADISSP–PPP1CA interaction may represent a previously unexplored vulnerability for disrupting oncogenic NF‐κB signaling in HCC.

Beyond regulating tumor intrinsic signaling, our study establishes ADISSP as a key mediator of tumor microenvironment remodeling. Through NF‐κB‐dependent transcriptional activation of SLC16A3 and CCL20, ADISSP coordinates metabolic adaptation and immune suppression through complementary mechanisms. SLC16A3 enhances glycolytic activity and lactate secretion, which promotes STAT3 activation and M2‐like macrophage polarization, whereas CCL20 facilitates macrophage recruitment into the tumor microenvironment. Consequently, ADISSP‐driven metabolic alterations actively reshape immune cell behavior and contribute to the establishment of an immunosuppressive niche. These findings provide mechanistic insight into the intimate connection between cancer metabolism and immune regulation and suggest that targeting metabolic‐immune coupling may represent an effective therapeutic strategy.

Our findings further demonstrate that ADISSP inhibition enhances the response to anti‐PD‐1 plus lenvatinib therapy by converting an immunosuppressive tumor microenvironment into a more immune‐permissive state. ADISSP depletion reduced M2 macrophage infiltration while increasing CD86(+) macrophages and cytotoxic CD8(+) T cells infiltration. Although additional studies are required to develop clinically applicable ADISSP‐targeting approaches and validate their therapeutic efficacy in patients, our study establishes ADISSP as a potential therapeutic target for enhancing immunotherapy sensitivity in HCC.

## 4 Materials and Methods

### 4.1 Cells and Cell Culture

The Huh7 (RRID: CVCL_0336), Hep3B (RRID: CVCL_0326), PLC/PRF/5 (RRID: CVCL_0485), THP‐1 (RRID: CVCL_0006), Hepa1‐6 (RRID: CVCL_0327), and the embryonic kidney cell line HEK‐293T (RRID: CVCL_0063) cell lines were authenticated through STR genotyping, showing no contamination and correct strain. Huh7, MHCC97H, Hepa1‐6, and PLC/PRF/5 cells were cultured in high‐glucose DMEM supplemented with 10% foetal bovine serum (Jet Biofil), 100 IU/mL penicillin, and 100 µg/mL streptomycin. Hep3B cells were maintained in MEM‐alpha supplemented with 10% foetal bovine serum, 100 IU/mL penicillin, and 100 µg/mL streptomycin. THP‐1 cells were cultured in RPMI 1640 supplemented with 10% foetal bovine serum, 100 IU/mL penicillin, and 100 µg/mL streptomycin. All the cell lines were cultured in 25 cm^2^ polystyrene flasks at 37°C in a humidified atmosphere containing 5% CO_2_.

### 4.2 Patients, Follow‐Up, and Tissue Microarrays

These studies were conducted in compliance with the ethical guidelines approved by the Ethics Committee of Qilu Hospital, Shandong University, with written informed consent obtained from all participants. A tissue microarray (TMA) was prepared by Shanghai Outdo Biotech Company from archived samples from 198 anonymized hepatocellular carcinoma (HCC) patients. Detailed clinicopathological data and follow‐up protocols are provided in Tables S4–S6.

### 4.3 RNA Sequencing (RNA‐seq) and Analysis of RNA‐Seq Data

Total RNA was extracted from HCC cell lines using TRIzol reagent (Invitrogen). The RNA concentration was determined using NanoDrop spectrophotometry (Thermo Scientific), and RNA integrity was evaluated using an Agilent 2100 Bioanalyzer with an RNA 6000 kit. Sequencing libraries were constructed using Illumina's TruSeq RNA Library Prep Kit and then quantified by qPCR, normalized, pooled, and sequenced on an Illumina HiSeq 4000 platform (RRID: SCR_016386). Differentially expressed genes were calculated by DESeq2 (RRID: SCR_000154). All experiments were performed with three biological replicates per group (Bioprofile Technologies Corporation, Shanghai, China).

### 4.4 ChIP‒qPCR

Chromatin immunoprecipitation (ChIP) was performed using a ChIP Assay Kit in accordance with the manufacturer's protocol. The cells were crosslinked with 1% formaldehyde, quenched with glycine, and lysed to extract chromatin. The chromatin was sheared by sonication into 200–500‐bp fragments. Immunoprecipitation was carried out overnight at 4°C using a specific antibody against the target protein. Protein‒DNA complexes were collected using protein A/G beads, washed, and eluted. The cross‐links were reversed, and the DNA was purified. qPCR was performed using primers specific to the target genomic region. The data were normalized to the input DNA and analysed using the ΔΔCt method. All the primer sequences that were used for qPCR are listed in Table S1.

### 4.5 qRT‐PCR Assay

Total RNA was extracted from tissues and HCC cell lines by using TRIzol reagent (15596026, Invitrogen, USA) according to the manufacturer's instructions. mRNA was reverse transcribed to cDNA using a PrimeScript RT Reagent Kit with gDNA Eraser (RR047A, Takara, Japan) according to the manufacturer's instructions. Quantitative PCR (qPCR) was subsequently performed with BlazeTaq qPCR Mix (QP031‐01, GeneCopoeia, USA). Expression was measured in triplicate in a Bio‐Rad CFX Connect Real‐Time System and normalised to human ACTB expression. The number of independent experiments was carried out three times. All the primer sequences that were used for qPCR are listed in Table S1.

### 4.6 Western Blot (WB) Analysis

Cells were lysed using RIPA buffer (R0020, Solarbio, China) supplemented with protease and phosphatase inhibitors. Protein concentration was determined using an Enhanced BCA protein assay kit (P0010, Beyotime, China). Equal amounts of protein were separated by SDS‐PAGE and transferred onto PVDF membranes. The membranes were blocked with 5% skim milk in TBST and incubated overnight at 4°C with primary antibodies, which are described in Table S2. After washing, membranes were incubated with HRP‐conjugated secondary antibodies for 1 h at room temperature. Protein bands were visualized using an ECL detection system, and the relative protein expression levels were quantified using ImageJ software.

### 4.7 Immunohistochemistry (IHC) Staining

Paraffin‐embedded tissue sections were deparaffinized, rehydrated, and subjected to antigen retrieval using citrate buffer (pH 6.0) in a microwave. After blocking endogenous peroxidase activity with 3% H_2_O_2_, sections were incubated with primary antibodies overnight at 4°C, as described in Table S2. After washing, sections were incubated with HRP‐conjugated secondary antibodies, followed by visualization using DAB substrate. Hematoxylin was used for counterstaining, and images were captured under a microscope.

### 4.8 Immunofluorescence (IF) Staining

Cells were seeded onto coverslips and fixed with 4% paraformaldehyde, followed by permeabilization with 0.3% Triton X‐100. After blocking with 5% BSA, cells were incubated with primary antibodies overnight at 4°C, which are described in Table S2, then followed by incubation with fluorescently labeled secondary antibodies. DAPI was used for nuclear staining. Fluorescence images were captured using a confocal microscope.

### 4.9 Cell Counting Kit‐8 (CCK‐8) Assay

Cells were seeded into 96‐well plates and cultured under the indicated conditions. At the designated time points, 10 µL of CCK‐8 reagent was added to each well and incubated for 1–2 h at 37°C. The absorbance at 450 nm was measured using a microplate reader to assess cell viability.

### 4.10 Transwell Migration and Invasion Assay

For the migration assay, cells were suspended in serum‐free medium and seeded into the upper chamber of a Transwell insert. The lower chamber contained complete medium with 10% FBS as a chemoattractant. After incubation for 24 h, non‐migrated cells were removed, and migrated cells on the lower surface were fixed with methanol, stained with crystal violet, and counted under a microscope. For the invasion assay, the upper chamber was pre‐coated with Matrigel before seeding the cells.

### 4.11 Colony Formation Assay

Cells were seeded in 6‐well plates at a low density (500–1000 cells per well) and cultured for 10–14 days. Colonies were fixed with methanol, stained with 0.5% crystal violet, and counted using ImageJ software. The colony formation rate was calculated as the number of colonies divided by the number of seeded cells.

### 4.12 EdU Proliferation Assay

The 5‐ethynyl‐20‐deoxyuridine (EdU) incorporation assay was performed using an EdU assay kit (Beyotime Biotechnology). Briefly, HCC cells were seeded in 24‐well plates at 1 × 10^5^ cells/well for 24 h. Then, 10 µm EdU reagent was added to each well and incubated for 2 h at 37 °C. Finally, the HCC cells were photographed using a fluorescence microscope (Nikon, Japan), and the percentage of EdU‐positive cells was calculated.

### 4.13 Flow Cytometry Analysis (Cell Death Assay)

SYTOX Green was used to analyse cell death. The percentage of stained cells was quantified via a CytoFLEX flow cytometer (Beckman Coulter, Brea, CA, USA) and a fluorescence microscope (Nikon, Japan).

### 4.14 Infection Experiment

Lentiviral constructs expressing short hairpin RNAs (shRNAs) and CMV promoter sequences were purchased from GeneChem (Shanghai, China) and were used for stable knockdown and overexpression in HCC cells, respectively. After 48 h, infected cells were cultured in a medium supplemented with puromycin (5 µg/mL, Solarbio, China) for 1 week to identify clones with stable expression. The infection sequences are listed in Table S3.

### 4.15 Co‐Culture Assay

In a Transwell co‐culture system, HCC cells were seeded in the upper chamber, while differentiated macrophages (derived from THP‐1 monocytes treated with 100 nm PMA for 48 h) were seeded in the lower chamber. Both cell types were cultured in RPMI‐1640 or DMEM supplemented with 10% FBS at 37°C with 5% CO_2_. This setup allowed for the exchange of soluble factors between HCC cells and macrophages without direct cell contact. After 24–48 h of co‐culture, cells or conditioned media from either chamber were collected for downstream analyses, such as cytokine profiling, gene expression, or functional assays.

### 4.16 Enzyme‐Linked Immunosorbent Assay (ELISA)

Cell culture supernatants or serum samples were collected and analysed using an ELISA kit in accordance with the manufacturer's instructions. Briefly, samples were added to precoated ELISA plates and incubated with specific detection antibodies. After washing, HRP‐conjugated secondary antibodies were added, followed by the addition of substrate solution. The reaction was stopped with a stop solution, and the absorbance was measured at 450 nm using a microplate reader. Human CCL20, TGFβ1 and IL‐10 ELISA kits (Abclonal, China) were used to determine the concentrations of CCL20, TGF‐β1 and IL‐10 in the supernatant in accordance with the manufacturer's protocol.

### 4.17 ATP Assay

The ATP assay was performed using the ATP Assay Kit (Biyuntian Biotechnology) according to the manufacturer's instructions. Briefly, cells were seeded in a 96‐well plate and treated under specified experimental conditions. After treatment, the cells were washed twice with PBS to remove residual medium. Cell lysis was achieved by adding 100 µL of lysis buffer to each well, followed by incubation at room temperature for 10 min with gentle shaking. The lysates were then centrifuged at 12 000 × g for 5 min at 4°C to remove cell debris. For ATP detection, 50 µL of the supernatant from each sample was transferred to a white opaque 96‐well plate, and 50 µL of ATP working solution (prepared by mixing the substrate and detection buffer provided in the kit) was added to each well. The luminescence signal was immediately measured using a microplate reader. A standard curve was generated using serial dilutions of the ATP standard solution to quantify ATP levels in the samples. Data were normalized to protein concentration or cell number for accurate comparison. This method provided a sensitive and reliable measurement of intracellular ATP levels.

### 4.18 ROS Assay, C11‐BODIPY 581/591 Assay, MDA Assay

The total intracellular ROS level was measured via the DCFH‐DA Kit with DCFH‐DA as the fluorescent probe. The MDA Test Kit and C11‐BODIPY 581/591 were used to measure lipid ROS levels. The concentrations of erastin and ferrostatin‐1 are 20 µM and 10 µm, respectively.

### 4.19 Iron Assay

The intracellular ferrous iron (Fe^2+^) level was determined using an iron assay kit (ab83366, Abcam) according to the manufacturer's instructions. The cells were seeded in a 10 cm^2^ plate (5 × 10^6^ cells per plate). The cells were collected and washed in ice‐cold PBS, homogenized in 5× volumes of iron assay buffer on ice, and then centrifuged (13,000 ×g, 10 min) at 4°C to remove insoluble material. The supernatant was collected, an iron reducer was added to each sample before mixing, and the mixture was incubated at room temperature for 30 min. Then, 100 µL of the iron probe was added to each sample, mixed, and incubated for 1 h at room temperature in the dark. The absorbance at 593 nm was measured immediately using a colorimetric microplate reader.

### 4.20 Lactate Assay

The lactate assay was performed using the Lactate Assay Kit (Solarbio) according to the manufacturer's instructions. Cells were seeded in a 6‐well plate and treated under specified experimental conditions. After treatment, the culture medium was collected, and cells were washed twice with PBS. Cells were then lysed using 200 µL of lysis buffer per well and incubated on ice for 10 min. The lysates were centrifuged at 12 000 × g for 10 min at 4°C to remove debris. For the assay, 50 µL of the supernatant or medium was transferred to a 96‐well plate and mixed with 50 µL of lactate assay buffer. Next, 10 µL of enzyme working solution (prepared by mixing lactate oxidase and peroxidase provided in the kit) was added to each well, followed by incubation at 37°C for 30 min in the dark. The absorbance was measured at 570 nm using a microplate reader. A standard curve was generated using lactate standard solutions to quantify lactate levels. Data were normalized to protein concentration or cell number for accurate comparison. This method provided a sensitive and reliable measurement of lactate levels in cells.

### 4.21 Seahorse Assay

We plated HCC control and knockdown cells at a density of 10 000 cells per well in an Agilent Seahorse XF96 cell culture microplate. Cellular metabolic activity was assessed using the Seahorse XF96 Extracellular Flux Analyzer (Agilent Technologies). For mitochondrial function analysis, the oxygen consumption rate (OCR) was measured following sequential injection of oligomycin (1 µm), FCCP (1 µm), and rotenone/antimycin A (1 µm each). Glycolytic function was evaluated by measuring the extracellular acidification rate (ECAR) after administration of glucose (1 µm), oligomycin (1 µm), and 2‐deoxyglucose (1 µm). All measurements were normalized to total protein content and analyzed using Agilent Wave software according to the manufacturer's protocols.

Glycolytic parameters were calculated according to the Agilent Seahorse XF Glycolysis Stress Test algorithm. Glycolysis was calculated as the maximum ECAR measured after glucose injection and before oligomycin injection minus the last ECAR measurement before glucose injection. Glycolytic capacity was calculated as the maximum ECAR following oligomycin injection minus the last ECAR measurement before glucose injection. Glycolytic reserve was calculated as glycolytic capacity minus glycolysis. The decrease in ECAR following 2‐DG injection was used to confirm that extracellular acidification was predominantly glycolysis‐dependent.

For the Mito Stress Test, non‐mitochondrial respiration was defined as the minimum OCR following rotenone/antimycin A injection. Basal respiration was calculated as the last OCR measurement before oligomycin injection minus non‐mitochondrial respiration. ATP‐linked respiration was calculated as the last OCR measurement before oligomycin injection minus the minimum OCR following oligomycin injection. Maximal respiration was calculated as the maximum OCR following FCCP injection minus non‐mitochondrial respiration, and spare respiratory capacity was calculated as maximal respiration minus basal respiration.

### 4.22 Immunohistochemistry (IHC) Staining

Paraffin‐embedded tissue sections were deparaffinized, rehydrated, and subjected to antigen retrieval using citrate buffer (pH 6.0) in a microwave. After blocking endogenous peroxidase activity with 3% H_2_O_2_, sections were incubated with primary antibodies overnight at 4°C, which described in Table S2. After washing, sections were incubated with HRP‐conjugated secondary antibodies, followed by visualization using DAB substrate. Hematoxylin was used for counterstaining, and images were captured under a microscope.

### 4.23 Infection Experiment

Lentiviral constructs expressing short hairpin RNAs (shRNAs) and CMV promoter sequences were purchased from GeneChem (Shanghai, China) and were used for stable knockdown and overexpression in HCC cells, respectively. After 48 h, infected cells were cultured in a medium supplemented with puromycin (5 µg/mL, Solarbio, China) for 1 week to identify clones with stable expression. The infection sequences are listed in Table S3.

### 4.24 Mice

All animal studies were conducted in compliance with ethical guidelines and were approved by the Ethics Committee of Qilu Hospital of Shandong University (DWLL‐2024‐320). Male BALB/c nude mice (5 weeks old) were purchased from Beijing Vital River Laboratory Animal Technology Co., Ltd. To establish subcutaneous HCC models, HCC cells transfected with lentiviral vectors were injected subcutaneously into the left axilla of nude mice. Tumor length and width were regularly measured using calipers, and tumor volume was calculated using the formula: Volume = (Length × Width^2^) / 2.

For orthotopic HCC models, 5‐week‐old C57BL/6 mice were purchased from Beijing Vital River Laboratory Animal Technology Co., Ltd. Plasmids were delivered via tail intravenous injection to induce hepatic tumor formation. Drugs were administered via intragastric gavage.

These models serve as valuable platforms for evaluating tumor growth dynamics, drug efficacy, and the underlying therapeutic mechanisms in vivo. All animal procedures were performed in accordance with institutional animal care and use guidelines.

### 4.25 Flow Cytometry Analysis of CD206

THP‐1 monocytes were differentiated into macrophages by treatment with 100 nm PMA for 48 h. Differentiated macrophages were harvested, washed with PBS, and stained with an anti‐CD206 antibody conjugated to a fluorescent dye for 30 min at 4°C in the dark. The cells were then washed twice with PBS to remove unbound antibodies and resuspended in 500 µL of PBS. Flow cytometry was performed using a flow cytometer, and the data were analysed using appropriate software. CD206 expression was quantified as the mean fluorescence intensity (MFI) or percentage of positive cells.

### 4.26 Bioinformatics and Statistical Analysis

Publicly available transcriptomic and clinical data used in this study were obtained from The Cancer Genome Atlas (TCGA) and the Gene Expression Omnibus (GEO), as described in the corresponding bioinformatics analyses. Differential expression analysis and data visualization were performed using R software (version 4.0.3) with the ggplot2 package (version 3.3.2). For experimental data, quantitative values were normalized to the corresponding control group or internal reference where applicable. No data points were excluded unless otherwise specified.

Data are presented as mean ± standard deviation (SD). Unless otherwise indicated, in vitro experiments were independently repeated at least three times. The exact sample size (*n*) for each experiment, animal study, and clinical analysis is provided in the corresponding figure legends. For comparisons between two independent groups, an unpaired two‐tailed Student's *t*‐test was used when the assumptions of approximate normality and homogeneity of variance were met. For comparisons involving more than two groups, one‐way analysis of variance (ANOVA) followed by Tukey's multiple‐comparisons test was used. Two‐way ANOVA was applied when two independent experimental factors were analyzed simultaneously. Categorical variables were analyzed using the chi‐square test. Correlations between protein expression levels in tissue microarrays were assessed using Spearman's correlation analysis. OS and RFS were estimated using the Kaplan–Meier method and compared using the log‐rank test. All statistical tests were two‐sided, and *p* < 0.05 was considered statistically significant. Statistical analyses were performed using IBM SPSS Statistics version 20.0 and GraphPad Prism version 11.0.

Other details regarding the materials and methods are described in the Information. The reagents used in this study are listed in the Supplementary Information.

## Author Contributions

**Tuan‐Song Zhang**: Writing – review & editing, Writing – original draft, Visualization, Validation, Software, Formal analysis. **Chen Xiong**: Data curation, Methodology. **Chen Yang**: Data curation, Methodology. **Yu‐Chuan Yan**: Formal analysis. **Jie Li**: Formal analysis. **Rui‐Zhe Li**: Formal analysis. **Guo‐Qiang Pan**: Formal analysis. **Jie Zou**: Conceptualization. **Xiao‐Lu Zhang**: Conceptualization. **Tao Li**: Supervision, Conceptualization. **Zao‐Ru Dong**: Writing – original draft, Visualization, Validation, Supervision, Funding acquisition, Conceptualization.

## Funding

This work was supported by grants from the National Natural Science Foundation of China (Grant No. 82172647), the Taishan Scholars Program of Shandong Province (Grant No. tsqn202312338), and Shandong Provincial Natural Science Foundation (Grant No. ZR2023QH368).

## Ethics Statement

This study was approved by Ethics Committee of Qilu Hospital, Shandong University (Approval No. QLCR20250662). Informed consent was obtained and accepted by all of the patients before enrollment. All methods were performed according to relevant guidelines and regulations. All animal experiments were approved by the Animal Care and Use Committee of Shandong University (DWLL‐2024‐320). All methods are reported in accordance with ARRIVE guidelines for the reporting of animal experiments.

## Consent

The author has nothing to report.

## Conflicts of Interest

The authors declare no conflicts of interest.

## Supporting information




**Supporting File**: advs78582‐sup‐0001‐SuppMat.docx.

## Data Availability

The data used to support the findings of this study are included and available within the article.
