# Supplementary material for: ADISSP Drives Metabolic Reprogramming and Immune Suppression to Regulate Hepatocellular Carcinoma Progression and Immunotherapy Sensitivity
Source: Adv Sci (Weinh). 2026 Nov 10:e78582. Online ahead of print. doi: 10.1002/advs.78582 (PMC13752238; doi:10.1002/advs.78582)
Supplement: Supplementary file 1 [file ADVS-9999-e78582-s001.docx]

Supplementary Figures

Figure S1. The relationship between ADISSP expression and HCC gene mutation/other HCC subtypes

(A) Comparison of ADISSP expression between ARID1A -mutant and ARID1A -wild-type tumors, CTNNB1-mutant and CTNNB1-wild-type tumors, TP53-mutant and TP53-wild-type tumors. Statistical significance was assessed using t-test; ns, not significant. (B) ADISSP expression in HCV-associated HCC and non-tumor liver tissues from the GSE17856 cohort. (C) ADISSP expression in alcohol-associated/non-viral HCC and non-tumor liver tissues from the GSE59259 cohort. P values are indicated in the plots; ns, not significant. (D) ADISSP expression in NASH/NAFLD-associated HCC and non-tumor liver tissues from the GSE99807, GSE164760 and GSE192959 cohort.

**Figure S2. ADISSP regulates HCC progression in vitro**

(A) Western blot (WB) analysis was performed to assess the basal ADISSP expression in several HCC cell lines. (B, C) qRT-PCR and WB confirmed the efficiency of ADISSP knockdown or overexpression. Mean ± SD, *n* = 3, Student's *t*‐test, ^*^ *p* < 0.05, ^**^ *p* < 0.01, ^***^ *p* < 0.001. (D) Transwell assays demonstrated the effects of ADISSP knockdown or overexpression on the migration and invasion abilities of HCC cells. Mean ± SD, *n* = 3, Student's *t*‐test, ^*^ *p* < 0.05, ^**^ *p* < 0.01, ^***^ *p* < 0.001. (E) Colony formation assay demonstrating the clonogenic ability of HCC cells. (F) CCK-8 assay showing the effect of ADISSP expression on HCC cell proliferation. mean ± SD, *n* = 4, two‐way ANOVA, ^*^ *p* < 0.05, ^**^ *p* < 0.01, ^***^ *p* < 0.001. (G) An EdU incorporation assay was performed to evaluate the role of ADISSP expression in HCC cell proliferation. Mean ± SD, *n* = 3, Student's *t*‐test, ^*^ *p* < 0.05, ^**^ *p* < 0.01, ^***^ *p* < 0.001.

Figure S3. ADISSP activates NF-κB signaling to transcriptionally upregulate the expression of SLC16A3 and CCL20 in HCC

 (A) Venn diagram showing the DEGs between Huh7-shADISSP and Hep-3B-shADISSP cells compared to their respective control cells. (B) IF staining showed the change in p65. Scale bars, 20 µm. (C-F) CCK-8 and Transwell assays were used to determine the effect of TNF-α (10 nM, with DMSO used as a control) or si P65 on the proliferation，migration and invasion abilities of HCC cells. (G) Venn diagram showed the overlap of DEGs identified from the FIMO_JASPAR, knockTF, ChIP_Atlas, hTFtarget and RNA-seq data. (H) qRT-PCR analysis of SLC16A3 and CCL20 expression in HCC cells. (I) IHC staining for SLC16A3 and CCL20 in mouse xenograft tumours. Scale bars, 20 µm. (J) Correlation analysis of ADISSP expression and SLC16A3/ CCL20 expression in the TMA data. (K) Kaplan–Meier survival analyses demonstrated significant prognostic associations for the combined expression of ADISSP with SLC16A3 or CCL20 in HBV-related HCC patients.

**Figure S4. SLC16A3 mediates ADISSP-dependent metabolic reprogramming in HCC cells** (A) Quantification of glycolysis, glycolytic capacity, and glycolytic reserve derived from the ECAR measurements. Mean ± SD, *n* = 3, two‐way ANOVA, ^*^ *p* < 0.05, ^**^ *p* < 0.01, ^***^ *p* < 0.001. (B) Quantification of basal respiration, ATP-linked respiration, maximal respiration, and spare respiratory capacity derived from the OCR measurements. Mean ± SD, *n* = 3, two‐way ANOVA, ^***^ *p* < 0.001. (C, D) Representative images and quantification of Transwell migration and invasion assays in ADISSP-KD HCC cells with SLC16A3 overexpression and in ADISSP-OE HCC cells with SLC16A3 knockdown. Mean ± SD, *n* = 3, two‐way ANOVA, ^**^ *p* < 0.01, ^***^ *p* < 0.001. (E, F) CCK-8 assays was used to evaluate the cell viability. Mean ± SD, *n* = 3, two‐way ANOVA, ^**^ *p* < 0.01, ^***^ *p* < 0.001. (G) Representative images and quantification of EdU incorporation assays assessing cell proliferation. Mean ± SD, *n* = 3, two‐way ANOVA, ^***^ *p* < 0.001. (H) Quantification of glycolysis, glycolytic capacity, and glycolytic reserve derived from the ECAR measurements in the indicated rescue groups. Mean ± SD, *n* = 3, two‐way ANOVA, ^***^ *p* < 0.001. (I) Quantification of basal respiration, ATP-linked respiration, maximal respiration, and spare respiratory capacity derived from the OCR measurements in the indicated rescue groups. Mean ± SD, *n* = 3, two‐way ANOVA, ^***^ *p* < 0.001.

**Figure S5. SLC16A3 mediates ADISSP-induced ferroptosis regulation in HCC** (A) Statistical analysis of ROS in HCC cells. Mean ± SD, *n* = 3, Student's *t*‐test, ^***^ *p* < 0.001. (B) Correlation heatmap of gene expression in HBV-related HCC. N=60. (C) Statistical analysis of the lipid ROS fluorescence signal in HCC cells. mean ± SD, *n* = 3, two‐way ANOVA, ^***^ *p* < 0.001. (D) Lipid peroxidation was measured by BODIPY™ 581/591 C11 staining. Scale bars, 100 µm. (E) FerroOrange and MitoTracker costaining demonstrated enhanced Fe^2+^ accumulation in shADISSP cells following erastin (20 µM) treatment. Scale bars, 20 µm. (F) Fe^2+^ levels in the indicated cells exposed to erastin (erastin: 20 μM). (G) Statistical analysis of the number of dead HCC cells. (H) Cell proliferation under the indicated conditions was assessed using CCK-8 assays. Mean ± SD, *n* = 3, two‐way ANOVA, ^***^ *p* < 0.001. (I) Statistical analysis of the lipid ROS fluorescence signal in HCC cells. (J) Quantification of cell death in RSL3-treated Hep3B and Huh7 cells. Cell death was assessed by SYTOX Green staining and flow cytometry. Mean ± SD, *n* = 3, two‐way ANOVA, ^***^ *p* < 0.001. (K) BODIPY™ 581/591 C11-based flow cytometric analysis of lipid ROS levels in erastin-treated Hep3B-KD and Huh7-KD cells with or without SLC16A3 overexpression. Mean ± SD, *n* = 3, Student's *t*‐test, ^***^ *p* < 0.001. (L) MDA levels in HCC cells exposed to erastin. Mean ± SD, *n* = 3, Student's *t*‐test, ^***^ *p* < 0.001. (M) Statistical analysis of the number of dead HCC cells. Mean ± SD, *n* = 3, two‐way ANOVA, ^***^ *p* < 0.001.

**Figure S6. ADISSP promotes immunosuppressive remodeling of the HCC tumor microenvironment** (A) Analysis of immune infiltration. (B) CD206, ARG1, IL-10, and TGF-β1 mRNA levels in macrophages were measured by qRT‒PCR. mean ± SD, *n* = 3, Student's *t*‐test, ^***^ *p* < 0.001. (C, D) Secretion of IL-10 and TGF-β in the supernatants of macrophages was detected by ELISAs. Mean ± SD, *n* = 4, Student's *t*‐test, ^***^ *p* < 0.001. (E) CD206, ARG1, CD86, and INOS mRNA levels in macrophages were measured by qRT‒PCR. Mean ± SD, *n* = 3, Student's *t*‐test, ^***^ *p* < 0.001. (F) Statistical analysis of CFSE proliferation of CD8+ T cells. (G) Xenograft tumors derived from Hepa1-6-KD cells were significantly smaller than those derived from the corresponding control cells. Mean ± SD, *n* = 6, Student's *t*‐test, ^***^ *p* < 0.001.

**Figure S7. SLC16A3-dependent lactate signaling and CCL20-mediated macrophage recruitment cooperatively establish an immunosuppressive tumor microenvironment** (A) CD206, ARG1, IL-10, and TGF-β mRNA levels in macrophages were measured by qRT‒PCR. Mean ± SD, *n* = 3, Student's *t*‐test, ^***^ *p* < 0.001. (B) Secretion of IL-10 in the supernatants of macrophages was detected by ELISAs. Mean ± SD, *n* = 4, Student's *t*‐test, ^***^ *p* < 0.001. (C) Secretion of TGF-β in the supernatants of macrophages was detected by ELISAs. Mean ± SD, *n* = 3, Student's *t*‐test, ^**^ *p* < 0.01, ^***^ *p* < 0.001. (D) CD206, ARG1, IL-10, and TGF-β mRNA levels in macrophages were measured by qRT‒PCR. Mean ± SD, *n* = 3, Student's *t*‐test, ^**^ *p* < 0.01, ^***^ *p* < 0.001, ^****^ *p* < 0.0001. (E, F) Flow cytometric analysis and quantification of CD206 expression in THP-1-derived macrophages exposed to conditioned medium from ADISSP-depleted HCC cells with or without L-lactic acid (5 mM) supplementation. Mean ± SD, *n* = 3, Student's *t*‐test, ^***^ *p* < 0.001. (G, H) ELISA analysis of TGF-β and IL-10 levels in THP-1-derived macrophages exposed to the corresponding conditions. Mean ± SD, *n* = 4, Student's *t*‐test, ^***^ *p* < 0.001, ^****^ *p* < 0.0001. (I) Representative WB analysis of p-STAT3 and STAT3 in THP-1-derived macrophages cultured with CM from sh-ADISSP cells with L-LA or ADISSP-OE cells with SLC16A3 knockdown. (J) Measurement of the pH of conditioned media collected from control and Huh7 -KD and Hep3B -KD cells, as well as control and PLC/PRF/5-OE cells. Mean ± SD, *n* = 3, Student's *t*‐test, ^***^ *p* < 0.001.(K) WB analysis of p-STAT3 and total STAT3 in THP-1-derived macrophages exposed to the corresponding conditions. (L) Quantification of macrophage Transwell migration in response to conditioned medium from control or Hepa1-6 ADISSP-OE cells, with or without CCL20-neutralizing antibody or CCR6 antagonist 1. Mean ± SD, *n* = 4, Student's *t*‐test, ^***^ *p* < 0.001.

**Figure S8. ADISSP activates NF-κB signaling by suppressing PPP1CA-mediated IKKβ dephosphorylation**.

 (A) Co-IP assays demonstrated that PPP1CA protein could interact with IKKβ protein directly. (B) The changes in proliferation ability of HCC cells with ADISSP-KD after being treated with the recombinant wild-type ADISSP or the T147A/Y163A double mutant. Mean ± SD, *n* = 3, two‐way ANOVA, ^***^ *p* < 0.001. (C) Representative images and quantification of Transwell migration and invasion assays in ADISSP-KD HCC cells reconstituted with wild-type ADISSP or the T147A/Y163A double mutant. Mean ± SD, *n* = 3, two‐way ANOVA, ^***^ *p* < 0.001.

**Figure S9. Targeting ADISSP remodels the tumor immune microenvironment and enhances the therapeutic efficacy of combined anti-PD-1 and lenvatinib treatment**

(A-C) Statistics of tumor-infiltrating CD206(+) macrophages, iNOS(+) macrophages, and CD8(+) T cells in corresponding HCC cells.

Table S1. List of primer sequences

| Gene | Forward primer sequence | Reverse primer sequence |
| --- | --- | --- |
| Homo-β-actin | CATGTACGTTGCTATCCAGGC | CTCCTTAATGTCACGCACGAT |
| Homo-ADISSP | TGCCCGTCCCTGAAGGTTAT | ACCTTCGCAGGCTAGCAGTA |
| Homo-SLC16A3 | CTTCCTGCTCACCATCCTGG | AGAGGTAGACGGAGTAGGGC |
| Homo-CCL20 | TTATTGTGGGCTTCACACGG | TTGCGCACACAGACAACTTT |
| Homo-CD206 | GATTGCAGGGGGCTTATGGG | CGGACATTTGGGTTCGGGAG |
| Homo-ARG1 | TGACGGACTGGACCCATCTT | GGCTTGTGATTACCCTCCCG |
| Homo-IL10 | CCAGACATCAAGGCGCATGT | GATGCCTTTCTCTTGGAGCTTATT |
| Homo-TGFβ | GCAACAATTCCTGGCGATACC | ATTTCCCCTCCACGGCTCAA |
| Homo-CCL20 for ChIP | ACCTTCGCACCTTCCCAATATG | GTACACAGAAGGCGTGTTGCC |
| Homo-SLC16A3 for ChIP | GAGGTGCTTTGAGGGAGCTG | AGACAGGAGGACTTTGTGGTC |
| Mus- ADISSP | ACTCGGCACACAAAGAAGGT | ACACTTGACACCATCCAGCA |
| Mus-CD206 | ACGAGCAGGTGCAGTTTACA | ACATCCCATAAGCCACCTGC |
| Mus-ARG1 | TGTCCCTAATGACAGCTCCTT | GCATCCACCCAAATGACACAT |
| Mus-β-actin | GGCTGTATTCCCCTCCATCG | CCAGTTGGTAACAATGCCATGT |
| Mus-CD86 | AACTTACGGAAGCACCCACG | CTCCACGGAAACAGCATCTGAG |
| Mus-INOS | CGAAACGCTTCACTTCCAA | TGAGCCTATATTGCTGTGGCT |

Table S2. List of primary antibodies

| Name | Supplier | Cat no. | RRID |
| --- | --- | --- | --- |
| Rabbit anti-human ADISSP | Thermo Fisher | PA5-113406 | RRID: AB_2868139 |
| Rabbit anti-human SLC16A3 | Abcam | ab308528 | RRID: AB_3712325 |
| Mouse anti-human Ki-67 | Cell Signaling Technology | **#**9449 | RRID: AB_2715512 |
| Rabbit anti-human CCL20 | Abcam | Ab9829 | RRID: AB_308798 |
| Rabbit anti-human NF-Κb P65 | Cell Signaling Technology | **#**8242 | RRID: AB_10859369 |
| Rabbit anti-human Phospho-NF-Κb P65 | Cell Signaling Technology | #3033 | RRID: AB_331284 |
| Rabbit anti-human Phospho-IκBα | Cell Signaling Technology | #2859 | RRID: AB_561111 |
| Rabbit anti-human IκBα | Proteintech | 10268-1-AP | RRID: AB_2151423 |
| Rabbit anti-human IKKB | Proteintech | 15649-1-AP | RRID: AB_2122307 |
| Mouse anti-human PPP1CA | Proteintech | 67070-1-lg | RRID: AB_2882379 |
| Rabbit anti-human Phospho-IKKB (Ser176/180) | Cell Signaling Technology | #2697 | RRID:  AB_2079382 |
| Mouse anti-human CD206 | Thermo Fisher | 17-2069-42 | RRID: AB_2573182 |
| Mouse anti-human CD16 | Elabscience | E-AB-F1236A | RRID: AB_3712302 |
| Mouse anti-human CD32 | Elabscience | E-AB-F1075A | RRID: AB_10900288 |
| Rabbit anti-human CD206 | Cell Signaling Technology | #24595 | RRID: AB_2892682 |
| Rabbit anti-human CD4 | Cell Signaling Technology | #25229 | RRID: AB_2798898 |
| Rabbit anti-human CD8 | Cell Signaling Technology | #98941 | RRID: AB_2756376 |
| Mouse anti-human 4-HNE | HUABIO | HA601598 | RRID: not available |
| Rabbit anti-human CCR6 | ABIOWELL | AWA45439 | RRID: not available |
| Rabbit anti-human PCNA | Cell Signaling Technology | #13110 | RRID: AB_2636979 |
| Rabbit anti-human F4/80 | Cell Signaling Technology | #30325 | RRID: AB_2798990 |
| Mouse anti-human GPX4 | Proteintech | 67763-1-Ig | RRID: AB_2909469 |
| Rabbit anti-human CD86 | Cell Signaling Technology | #91882 | RRID: AB_2797422 |
| Anti-mouse iNOS Alexa Fluor™ 488, | eBioscience™ | 53-5920-82 | RRID: AB_2574423 |
| Anti-mouse CD206 PE | eBioscience™ | 12-2061-82 | RRID: AB_2637422 |
| Anti-mouse CD11b, Brilliant Violet™ 650 | eBioscience™ | 416-0112-82 | RRID: AB_2925670 |
| Anti-Mouse F4/80 PE/Cyanine7 | elabscience | E-AB-F0995H | RRID: AB_3102042 |
| Anti-Mouse CD45 PerCP/Cyanine5.5 | BioLegend | 157208 | RRID: AB_2860728 |
| anti-mouse CD3 PerCP/Cyanine5.5 | BioLegend | 100218 | RRID: AB_1595492 |
| anti-mouse CD8a Brilliant Violet 510 | BioLegend | 100752 | RRID: AB_2563057 |

| Gene | **Transfection Sequences** |
| --- | --- |
| Human-shADISSP-1 | CCUGAAGGUUAUAGUGUCATT |
| Human-shADISSP-2 | GCGCCGAGCUGGAAUACGATT |
| Mus-shADISSP | CAAUCGCUCUGUUCCGAGUCATT |
| Human-siP65 | GGAGCACAGATACCACCAAGA |
| Human-siSLC16A3 | GCAUUGAGACCCUGGUUCATT |
| Human-siPPP1CA | GACGCUACAACAUCAAACUTT |

Table S3. List of transfection sequences

Table S4. Statistics for ADISSP and clinicopathologic features in HBV-related HCC patients

| Clinicopathological feature | ADISSP expression | | | *χ2* | *P* value |
| --- | --- | --- | --- | --- | --- |
|  | Total | Low | High |  |  |
|  | 167 | 85 | 82 |  |  |
| Age (years) | | | | | |
| ≤50 | 102 | 47 | 55 | 1.9656 | 0.1609 |
| >50 | 65 | 38 | 27 |  |  |
| Gender | | | | | |
| Male | 151 | 75 | 76 | 0.50877 | 0.4757 |
| Female | 16 | 10 | 6 |  |  |
| Tumour diameter | | | | | |
| ≤5 cm | 80 | 48 | 32 | 4.4152 | 0.03562 |
| >5 cm | 87 | 37 | 50 |  |  |
| Vascular invasion | | | | | |
| None | 154 | 81 | 73 | 1.4955 | 0.2214 |
| Yes | 13 | 4 | 9 |  |  |
| Tumour capsule | | | | | |
| Yes | 86 | 52 | 34 | 5.7282 | 0.01669 |
| None | 81 | 33 | 48 |  |  |
| Tumour number | | | | | |
| 1 | 132 | 75 | 57 | 7.738 | 0.005407 |
| ≥2 | 35 | 10 | 25 |  |  |
| AFP | | | | | |
| ≤400 ng/ml | 51 | 35 | 16 | 8.2414 | 0.004095 |
| >400 ng/ml | 111 | 50 | 66 |  |  |
| Tumour differentiation | | | | | |
| I-II | 94 | 48 | 46 | 0 | 1 |
| III-IV | 73 | 37 | 36 |  |  |
| Liver cirrhosis | | | | | |
| None | 26 | 12 | 14 | 0.098076 | 0.7542 |
| Yes | 141 | 73 | 68 |  |  |
|  |  |  |  |  |  |

Table S5. Univariate and multivariate analysis of OS in HBV-related HCC patients

| Variable | Univariate | | Multivariate | |
| --- | --- | --- | --- | --- |
|  | HR (95%Cl) | P value | HR (95%Cl) | P value |
| ADISSP level (high vs low) | 1.085 (1.065-1.105) | <0.001 | 1.087 (1.064–1.111) | <0.001 |
| Age (years) (≤50 vs >50) | 1.158 (0.789-1.700) | 0.454 | 1.270 (0.832–1.940) | 0.268 |
| Gender (male vs female) | 1.576 (0.732-3.392) | 0.245 | 2.689 (1.153–6.274) | 0.022 |
| Tumour diameter (cm) (≤5 vs >5) | 1.904 (1.293-2.803) | 0.001 | 1.649(1.077-2.525) | 0.021 |
| Vascular invasion (Yes vs None) | 4.002 (2.205-7.264) | <0.001 | 3.603(1.819-7.138) | <0.001 |
| Tumour number (≥2 vs 1) | 2.219 (1.449-3.397) | <0.001 | 1.373 (0.854–2.207) | 0.191 |
| AFP (ng/ml) (≤400 vs >400) | 1.652 (1.068-2.557) | 0.024 | 1.239 (0.772–1.987) | 0.374 |
| Tumour differentiation (III-IV vs I-II) | 0.951 (0.647-1.397) | 0.796 | 1.246 (0.831–1.868) | 0.288 |
| Liver cirrhosis (Yes vs None) | 1.545 (0.863-2.764) | 0.143 | 1.884(1.005-3.530) | 0.048 |

Table S6. Univariate and multivariate analysis of RFS in HBV-related HCC patients

| Variable | Univariate | | Multivariate | |
| --- | --- | --- | --- | --- |
|  | HR (95%Cl) | P value | HR (95%Cl) | P value |
| ADISSP level (high vs low) | 1.031 (1.006-1.058) | 0.017 | 1.029 (1.002-1.057) | 0.038 |
| Age (years) (≤50 vs >50) | 1.021 (0.613-1.700) | 0.936 | 1.062 (0.613-1.839) | 0.831 |
| Gender (male vs female) | 2.224 (0.697-7.095) | 0.177 | 2.573 (0.772-8.570) | 0.124 |
| Tumour diameter (cm) (≤5 vs >5) | 1.031 (0.627-1.694) | 0.905 | 0.999 (0.592-1.687) | 0.997 |
| Vascular invasion (Yes vs None) | 1.843 (0.658-5.167) | 0.245 | 1.998 (0.673-5.931) | 0.212 |
| Tumour number (≥2 vs 1) | 1.359 (0.720-2.563) | 0.344 | 1.055 (0.536-2.075) | 0.877 |
| AFP (ng/ml) (≤400 vs >400) | 1.760 (0.997-3.106) | 0.051 | 1.724 (0.957-3.106) | 0.070 |
| Tumour differentiation (III-IV vs I-II) | 0.819 (0.493-1.360) | 0.441 | 0.961 (0.570-1.620) | 0.882 |
| Liver cirrhosis (Yes vs None) | 1.660 (0.786-3.504) | 0.184 | 1.846 (0.846-4.030) | 0.124 |

Table S7. Clinical characteristics of 40 HBV-related HCC patients

| Characteristics | | ADISSP high level | ADISSP low level |
| --- | --- | --- | --- |
| Age (years) (≤ 57/> 57) | | 9/11 | 12/8 |
| Sex (male/female) |  | 18/2 | 16/4 |
| Child-Pugh classification (A/B) | | 19/1 | 19/1 |
| AFP (≤ 400/> 400 ng/ml) | | 6/14 | 6/14 |
| Tumor differentiation (I/II vs III/IV) | | 11/9 | 14/6 |
| Tumor biggest diameter (≤5/＞5cm) | | 6/14 | 9/11 |
| Number of tumor (≤2/≥ 3) | | 15/5 | 16/4 |
| Vascular Invasion (Yes vs None) | | 5/15 | 5/15 |
